# Rapid selection and identification of functional CD8^+^ T cell epitopes from large peptide-coding libraries

**DOI:** 10.1038/s41467-019-12444-7

**Published:** 2019-10-07

**Authors:** Govinda Sharma, Craig M. Rive, Robert A. Holt

**Affiliations:** 1 Michael Smith Genome Sciences Centre, British Columbia Cancer Agency, 675W 10th Ave, Vancouver, BC V5Z 1L3 Canada; 2 Department of Medical Genetics, University of British Columbia, C201 – 4500 Oak Street, Vancouver, BC V6H 3N1 Canada; 3 Department of Molecular Biology and Biochemistry, Simon Fraser University, SSB8166 – 8888 University Drive, Burnaby, BC V5A 1S6 Canada

**Keywords:** High-throughput screening, Lymphocyte activation, MHC class I, CD8-positive T cells

## Abstract

Cytotoxic CD8^+^ T cells recognize and eliminate infected or malignant cells that present peptide epitopes derived from intracellularly processed antigens on their surface. However, comprehensive profiling of specific major histocompatibility complex (MHC)-bound peptide epitopes that are naturally processed and capable of eliciting a functional T cell response has been challenging. Here, we report a method for deep and unbiased T cell epitope profiling, using in vitro co-culture of CD8^+^ T cells together with target cells transduced with high-complexity, epitope-encoding minigene libraries. Target cells that are subject to cytotoxic attack from T cells in co-culture are isolated prior to apoptosis by fluorescence-activated cell sorting, and characterized by sequencing the encoded minigenes. We then validate this highly parallelized method using known murine T cell receptor/peptide-MHC pairs and diverse minigene-encoded epitope libraries. Our data thus suggest that this epitope profiling method allows unambiguous and sensitive identification of naturally processed and MHC-presented peptide epitopes.

## Abstract (web summary)

High-throughput assays for TCR specificity are a bottleneck in understanding T cell immunity and harnessing it for medicine. Here the authors develop a functional screening method to identify T cell specificity in the natural context of peptide-MHC presentation, enabling detection of physiologically relevant T cell antigens from large libraries of peptide-coding sequences.

## Introduction

Identifying T-cell epitopes is a difficult task due to the complexity of antigen-specific T-cell activation. Contributing to this complexity are several factors. First, the number of unique short peptides that could possibly exist makes for an immense T-cell epitope space to be searched. Second, peptide-presenting major histocompatibility complex (MHC) molecules are encoded in humans by *HLA* genes that are polygenic and highly polyallelic, with different *HLA* alleles encoding MHC variants having distinct peptide-binding and T-cell receptor (TCR)-binding preferences. Third, variation in the intracellular expression level of antigenic proteins and biases in proteolytic processing also influence pMHC immunogenicity^1^. Finally, TCR/peptide-MHC (pMHC) interactions are transient^2^, promiscuous^3^, and, compared with epitope recognition by antibodies, relatively low-affinity^4^.

Various function-based and affinity-based methods of antigen screening are in current use^5^. In the function-based class of methods, candidate T-cell peptide are presented on target cell surfaces and tested for their ability to generate functional T-cell responses. These responses are then identified either by using a T cell-based read-out, such as cytokine release^6^, activation of an NFAT-linked reporter^7–9^, or, alternatively, monitoring destruction of antigen-presenting cells (APC) to measure functional T-cell activation^10^. These configurations all have in common the requirement to load target cell populations with individual candidate antigens and test each of them for T-cell recognition “one-by-one” in separate reactions. Pooling strategies can increase the search space, but would, subsequently, need to be laboriously deconvoluted^11–13^. Thus, functional cellular assays have yet to be scaled in a manner that could conceivably enable exhaustive screening of large sets of potential epitopes, such as those spanning an entire proteome.

In contrast to function-based screening assays, affinity-based methods such as single-chain MHC display^14–16^ or combinatorial/barcoded pMHC-multimer surface staining^17,18^ have been developed that seek to circumvent many of the limitations mentioned above. Although scalable, these methods bypass natural antigen processing, presentation, and T-cell activation, and rely solely on TCR/pMHC affinity as a proxy for T-cell recognition. Important biophysical parameters, including kinetic on/off rates, allosteric effects of the TCR on downstream signaling, pMHC half-life, and the action of co-receptors, are all known to be critical to the activation of T cells^19–24^, but are not taken into account in these techniques. Therefore, affinity-based methods may in some cases yield high-affinity epitopes that are physiologically irrelevant, while missing other epitopes that are of low-affinity but physiologically important^25^.

New methods that combine the strengths of the function-based approaches with the scalability of affinity-based approaches are crucial for further understanding T-cell biology. At present, TCR sequencing (TCR-seq) studies are routinely used to reveal millions of unique TCR α- and/or β-chains per individual and investigate the T-cell repertoires of dozens of individuals per study^26^. Indeed, the generation of large volumes of TCR-seq data in recent years has necessitated the creation of large public repositories containing on the order of 10^8^–10^9^ TCR sequences^27^. From these vast TCR-seq resources, significant data-mining efforts have been undertaken in order to understand patterns of TCR sequence convergence^28,29^ and identify particularly interesting TCR clonotypes. However, TCR-seq data do not provide any information regarding the specific antigenic determinants of these clonotypes. Moreover, the phenomenon of T cell cross-reactivity is now well-appreciated as an intrinsic property of the T-cell receptor^3^ and it is, therefore, now important to pursue rational screening of T-cell populations-of-interest against vast and unbiased libraries of peptides to reveal the landscape, or repertoire, of epitopes they recognize.

Our approach^30^ to conducting high-throughput function-based T-cell antigen discovery is based on a fundamentally different design from classic T-cell activity assays, wherein the main concept is the co-expression of candidate epitopes, encoded as minigenes in APC, and a reporter system that is intrinsic to the APC instead of the T cell. This configuration allows for targeted APC to become distinguishable from non-targeted APC in T-cell co-cultures and facilitate selective recovery of immunogenic antigen-bearing cells from irrelevant bystanders in the bulk population.

**Fig. 1:**
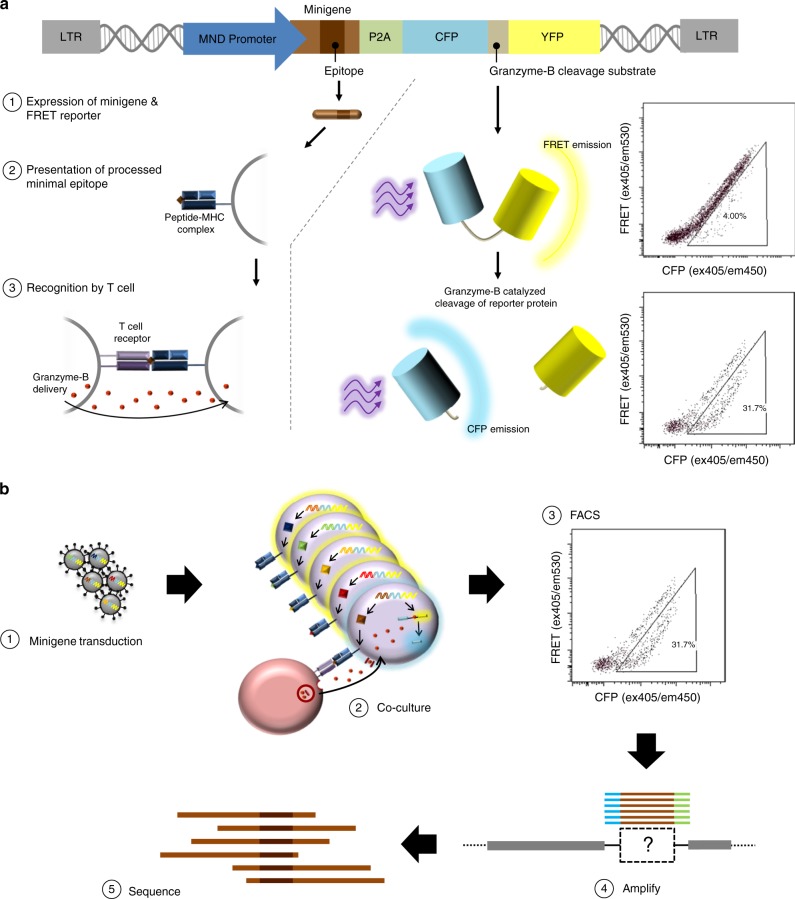
Summary of epitope-screening method. **a** In the absence of GZMB, the reporter protein emits a resting FRET signature when excited with violet light. Upon entry of GZMB to the cell, the reporter is cleaved. The loss of resting FRET signal combined with the rescue of free CFP signal results in a FRET-shift that can be monitored in FACS to isolate cells undergoing T-cell targeting. **b** Target cells are provided with libraries of lentivirally delivered short-peptide-coding minigene sequences and exposed to T cells of interest. Any targets recognized by T cells are then subject to GZMB loading and cleavage of their internally expressed FRET reporter. Cells carrying putatively antigenic minigenes are isolated by FACS, minigenes encoded within these cells are recovered by PCR, and the resultant amplicons are characterized by deep sequencing

To realize this design, we leverage the exquisite specificity of the granzyme–perforin pathway. In vivo, a cytotoxic T-cell (CTL) recognizing a target cell employs this pathway to deliver a class of proteases called granzymes, predominantly granzyme B (GZMB), into the target cell without entry into bystander cells^31^. Once inside a target cell, GZMB initiates an apoptotic cascade that leads to cell death. We constructed a reporter fusion protein consisting of cyan fluorescent protein (CFP) and yellow fluorescent protein (YFP) moieties separated by a peptide linker that also acts as a cleavage substrate for GZMB^32,33^. When fused, the CFP-YFP reporter protein produces Förster resonance energy transfer (FRET) signal while partially quenching CFP emission upon excitation with violet light. Cleavage of the fusion protein by GZMB causes a loss of FRET signal and concomitant rescue of free CFP signal. The resulting shift of cells carrying cleaved FRET reporter is easily distinguishable in FACS and, thus, allows for targeted cells to be isolated and recovered (Fig. 1a).

For use in T-cell epitope screening, large libraries of short-peptide-coding DNA minigene sequences are cloned into a lentiviral transfer plasmid alongside the GZMB-cleavable FRET reporter (Supplementary Fig. 1), which are used to generate lentiviral minigene libraries. Infection of sero-matched target cells with libraries is performed at a multiplicity of infection (MOI) that favors a single minigene per APC, and transduced target cells are co-incubated with expanded CTL populations-of-interest. After co-culture, FACS is conducted on the basis of FRET-shift status to isolate only target cells carrying putatively antigenic minigenes. Recovered minigenes are PCR-amplified and sequenced to reveal the epitopes eliciting reactivity from the screened CTL (Fig. 1b).

Here, we validate our FRET-shift FACS/deep amplicon-sequencing methodology with respect to efficacy, specificity, and kinetics of GZMB delivery in vitro, as well as the sensitivity of the approach in detecting bona fide antigens from large libraries of candidate peptide-coding sequences and the robustness of the assay to mixed input T-cell populations. We present this method as a tool for facilitating the study of cellular immunity, supplementing the existing T-cell epitope knowledge-base^34^, and guiding the development of novel T cell-based immunotherapies in the context of cancer, autoimmunity, and infectious disease.

## Results

### Efficient detection of GZMB-targeted cells

**Fig. 2:**
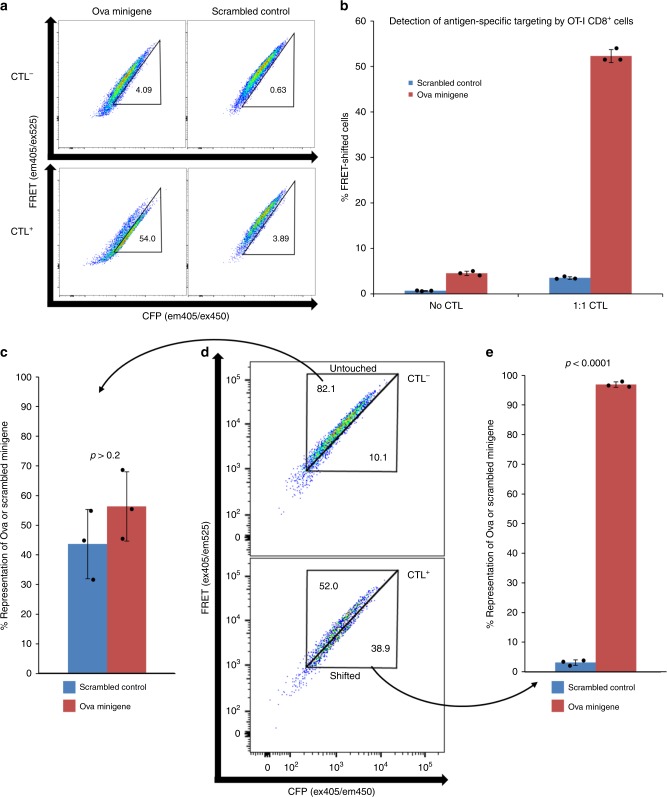
FRET-shift assay testing. **a**, **b** ID8 cells expressing either the Ova minigene fragment with native epitope intact (OVAL241-280) or the Ova minigene containing a scrambled epitope (OVAL257-264 SIINFEKL → LKNFISEI) were cultured with or without OT-I CD8^+^ T cells at a 1:1 ratio for 4 h. **a** Representative plots of FRET signal (ex405/em525) vs. CFP signal (ex405/em450) and (**b**) proportions of cells shifting into Targeted gate in all replicates (*n* = 3, underlaid bar chart and error bars denote mean ± SD) are shown. Significance was determined using an unpaired, one-tailed Student’s *t* test. Effect size was calculated as the difference of standardized means (Cohen’s effect size). **c**–**e** Ova minigene-expressing targets or scrambled control cells were combined at a 1:1 ratio to form a binary mixed target population. Mixed targets were co-incubated with OT-I CTL for 4 h at 1:1 effector:target ratio prior to FACS analysis in triplicate for all conditions (underlaid bar chart and error bars denote mean ± SD). Recovered cells were lysed, and genomic DNA was purified and used as a template for qPCR using a custom TaqMan assay. Significance was determined using an unpaired, one-tailed Student’s *t* test. Source data are provided as a Source Data file

We first sought to validate the GZMB-cleavable reporter gene as a useful tool for T-cell epitope identification. The murine ovarian cancer cell line, ID8, was used as APC for the well-characterized model TCR, OT-I^35^. ID8 cells were transduced with minigene constructs coding for a 40 amino acid stretch of the chicken ovalbumin protein (OVAL241-280) with either the intact OT-I minimal epitope (SIINFEKL), or a scrambled version of this epitope (LKNFISEI), at the center of this region. CD8^+^ T cells were expanded by anti-CD3/28 stimulation from splenocytes of the OT-I TCR-transgenic mouse^36^, and co-cultured with each target cell line separately. Flow-cytometric analyses of co-cultures indicated that SIINFEKL^+^ cells underwent significant (*p* < 0.0001 by unpaired, one-tailed Student’s *t* test) and substantial (Cohen’s *d* > 40) cleavage of their encoded reporter protein relative to the scrambled negative control. These data provide evidence that the FRET-shift assay described herein is capable of efficiently detecting target cells harboring the correct antigen (Fig. 2a, b).

### GZMB delivery to targeted cells is highly specific

Once the GZMB-sensitive FRET-shift assay was shown to be epitope-specific in homogenous target cell populations, we tested the specificity of the method in a mixed population. As GZMB is a soluble effector molecule released by CTL on recognition of cognate epitope, we considered the possibility that perforin and GZMB escaping from immunological synapses formed between a CTL and APC could potentially diffuse into irrelevant bystander cells and produce false-positive signal. To evaluate this, Ova minigene-expressing cells and scrambled control minigene-expressing cells were mixed in a 1:1 ratio and co-cultured with OT-I CTL. Upon completion of co-culture, cells were sorted according to the gating scheme shown in Fig. 2d. Transduced ID8 cells that FRET-shifted into the “Shifted” gate under T-cell pressure and cells in the “Untouched” gate of CTL^−^ controls, a sample of the base minigene-expressing population that was not altered by T-cell pressure, were both recovered and analyzed by genomic qPCR to determine the relative proportion of Ova minigenes and scrambled minigenes present in either population. After correcting for the average integrations/cell of each transduced ID8 cell line using qPCR (Supplementary Fig. 2), we found that > 95% of the cells captured in the Targeted gate expressed Ova minigenes (Fig. 2e, *p* < 0.0001 by unpaired, one-tailed Student’s *t* test), while the cells captured in the Untouched gate were found to remain in a roughly 1:1 ratio of Ova minigene-expressers to scrambled control-expressers as expected (Fig. 2c, *p* > 0.2, no significant difference by unpaired, one-tailed Student’s *t* test).

### Peak GZMB signal precedes apoptosis of target cells

Counterintuitively, the GZMB FRET-shift assay relies on isolating antigen-encoding target cells that have received a delivery of granzyme B from activated CTL and have initiated apoptosis. We sought, therefore, to investigate the kinetics of FRET-shift signal as it relates to the kinetics of apoptosis progression in our in vitro co-culture system and to establish a timeframe for safe-sorting. The OT-I CTL/Ova minigene model system was again employed. Four different effector:target ratios were set up and monitored for both % FRET-shifted and % propidium iodide (PI)-positive at ten different time points. FRET-shift signal was detectable as early as 1 h after initiation of CTL/target cell co-culture and steadily rose to a peak value after 6–8 h (Supplementary Fig. 3a). Signal from PI staining—indicative of loss of cell membrane integrity and cell death—was observed to spike 2–4 h after peak FRET-shift signal was attained (Supplementary Fig. 3b) before sharply declining as cells deteriorated due to apoptosis. Thus, there is a safe-sorting window during which APC that have received a granzyme hit can be recovered and DNA-extracted for epitope identification.

### Antigenic minigenes are detectable with high sensitivity

We next measured the sensitivity of this approach as defined as the limit of detection of a canonical test antigen when the antigen is present in a background minigene population in decreasing abundances. To explore this, we constructed and tested a diverse library of random minigenes. Degenerate oligonucleotides containing a stretch of 48 randomized bases were synthesized, amplified by PCR, and ligated into the minigene site of the FRET reporter-containing lentiviral transfer plasmid (Supplementary Fig. 1). Plasmid ligation products were transformed into electrocompetent bacteria and amplified on solid agar to obtain ≈1.8 × 10^6^ random minigene clones, from which plasmid DNA was isolated and used to generate lentivirus. Lentivirus was titered and transduced into ID8 cells at an MOI favoring one insertion event per cell to produce a random minigene-expressing target cell population (Supplementary Table 1).

Five separate cell populations were prepared for screening by combining Ova-minigene-expressing ID8 cells with random minigene-expressing ID8 cells in abundances ranging from 1:10 to 1:100,000. Doping random minigene-expressing cells with canonical minigene-expressing cells was done at the cellular level as opposed to spiking at the DNA level by combining different plasmid construct at varying ratios. The rationale for this was that, because of the need to undergo a lengthy purity sort to isolate a high-diversity random minigene-expressing population after viral transduction, it would be more efficient to prepare the library once and generate spiked populations from the base library population rather than to repeatedly perform intensive FACS preparations of DNA level-spiked libraries. We performed a spike-in method comparison study and confirmed that the method of minigene spiking did not significantly alter the detection of spiked sequences in downstream analyses (Supplementary Fig. 4).

**Fig. 3:**
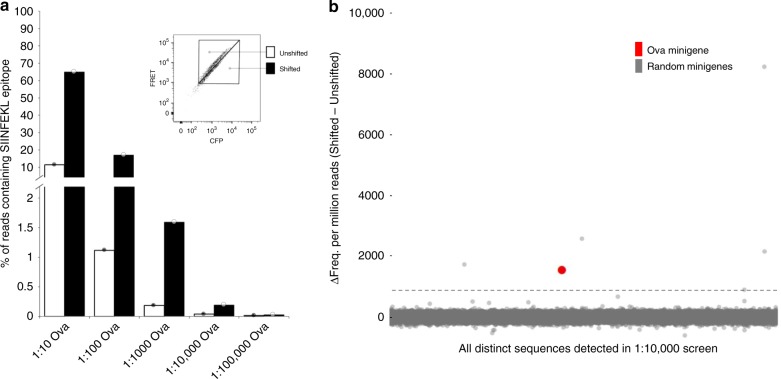
OT-I spiked library screening. Random minigene cell libraries spiked with Ova minigene-expressing cells were co-incubated with OT-I CTL at a 1:1 effector:target ratio for 4 h prior to FACS analysis. Recovered cells were lysed and integrated minigenes were amplified from genomic DNA using PCR primers specific for the conserved transgene region flanking the minigene site. Primers with indexed Illumina adapter tails were used for direct sequencing of amplicons on the Illumina MiSeq platform with 2 × 250 paired-end chemistry. **a** The percentage of reads detected in each gate that encoding the SIINFEKL epitope. Filled circles underlaid with white bars represent Unshifted gate; open circles underlaid with black bars represent Shifted gates. Each point represents one individual measurement. Source data are provided as a Source Data file. **b** The read count frequency of all unique sequences found in 1:10,000 spike-in sample expressed as the difference between the relative frequency in the Shifted gate and the Unshifted gate. The dashed line represents 10σ above the mean Δ relative abundance value

Each cell level-spiked cell population was co-cultured with OT-I CTL and sorted by FRET status to obtain Shifted and Unshifted gates for all spike-in levels (Fig. 3a inset). Gates were defined by establishing the boundaries of resting FRET signature on CTL^−^ control populations. Genomic DNA was isolated from each collected cell population and used as a template for PCR amplification of integrated minigenes using Illumina adapter-tailed primers for direct sequencing on the Illumina MiSeq platform. By analyzing the Δ relative abundances of Ova-minigene reads detected in the Shifted and Unshifted gates of each spiked library screen, we determined that at the 1:10, 1:100, and 1:1000 spike-in levels, the Ova-minigene was the most prevalent sequence detected in the Shifted gates and was considerably enriched relative to Unshifted populations (Fig. 3a). At 1:10,000, the Ova-minigene was no longer observed to be the most dominant enriched sequence, however, it was still in the top five most highly enriched minigenes and was detected at > 10 standard deviations (σ) above background (Fig. 3b). These data indicate that cells presenting relevant epitope are easily detectable even when present at a population frequency of 1:10,000, which is generally considered the signal detection cutoff of conventional assays^37^.

FRET-shift signal detection was also tested using a second mouse TCR/pMHC pair and an alternative host target cell line. We used cytotoxic T cells derived from the pmel-1 TCR-transgenic mouse line^38^, which are known to be stimulated by recognition of the hgp100(25–33) peptide, KVPRNQDWL, in the context of mouse H-2Kd. To this end, an hgp100 minigene sequence was constructed encoding the minimal epitope along with 16 amino acids of endogenous flanking sequence and inserted into the minigene-FRET lentiviral cassette in similar fashion to the Ova minigenes described above. After virus production, both hgp100 minigene and Ova minigene constructs were transduced into the EL4 cell line, a mouse T-cell lymphoma induced in the C57BL/6 strain. Co-cultures were performed using both OT-I CTL and pmel-1 TCR CTL against Ova minigene and hgp100 minigene-expressing EL4 cells, respectively. From these experiments, we observed that both a higher percentage of target cells were FRET-shifted in Ova minigene-expressing EL4 cells compared with their ID8 counterparts and that the resulting FRET-shift was larger in magnitude (Supplementary Fig. 5), which is potentially a reflection of the difference in MHC-I expression intrinsic to either cell line (Supplementary Fig. 6).

**Fig. 4:**
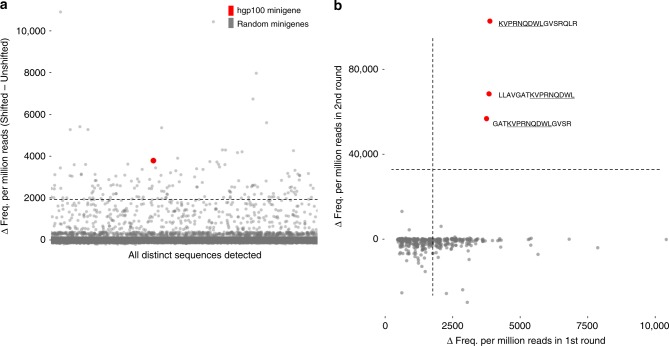
pmel-1 TCR screening against 1:10,000 hgp100 minigene-spiked random library. **a** Random minigene-expressing EL4 cell library spiked with hgp100 minigene-expressing EL4 cells at an abundance of 1:10,000 were co-incubated with pmel-1 TCR CTL at a 1:1 effector:target ratio for 4 h prior to FRET-shift FACS analysis. Upon recovery and sequencing of minigenes captured in Unshifted and Shifted gates, differences in relative abundance for all distinct minigene sequences were plotted. The dashed line represents 10 σ above the mean Δ relative abundance value. **b** The top 480 minigenes enriched in the primary screen of pmel-1 TCR CTL vs. a 1:10,000 hgp100-spiked random minigene library were synthesized as a ssDNA oligonucleotide pool and inserted into the pMND-silent-FRET lentiviral transfer plasmid. A pmel-1 TCR panning second-round viral library was produced and delivered into EL4 cells. Transduced and purified panning minigene-expressing EL4 cells were then co-cultured (1:1 E:T, 4 h) with freshly activated and expanded pmel-1 TCR CTL and sorted according to FRET-shift. After minigene recovery by PCR and amplicon sequencing, the relative abundances of each member of the panning library in the primary (*x*-axis) and secondary (*y*-axis) screens were plotted. The 16-mer encoding hgp100 minigenes that were included in the synthesized panning library are highlighted and labeled. Dashed lines denote 10σ above the mean Δ relative abundance value

We then determined that the sensitivity demonstrated in the OT-I/Ova system could be recapitulated in the pmel-1 TCR/hgp100 system by spiking hgp100 minigene-expressing EL4 cells into random minigene-expressing EL4 (Supplementary Table 2) cells at an abundance of 1:10,000 and co-culturing the mixed population with pmel-1 TCR CTL. Both the Shifted and Unshifted populations were isolated by FACS and used to prepare minigene amplicon libraries for Illumina sequencing. Raw reads were processed and analyzed as done previously with Ova-spiked libraries to reveal that hgp100 could, indeed, also be detected > 10σ above background (Fig. 4a).

### Strategies for refining putative hits

One does not have a priori knowledge of T-cell epitopes when performing unbiased epitope discovery. Here, we noted that a number of randomly encoded minigenes met the 10σ cutoff in addition to the spiked-in canonical minigenes (Figs. 3b,  4a). We, therefore, evaluated whether a subset of these sequences could contain bona fide epitopes by analyzing them with NetMHCpan^39^ to determine if they contained any predicted MHC-binding peptides and by aligning them to the GenBank nonredundant cds translation database by BLASTP^40^ to determine if they had any significant sequence similarity to known proteins (Supplementary Table 3). For this analysis, we selected the minigene sequences that were found to be more enriched than the known antigenic minigene: in each case, this was the top three from the OT-I/Ova condition and the top 15 from pmel-1 TCR/hgp100 condition.

None of the minigenes identified in the OT-I/Ova screen other than the spiked-in canonical Ova minigene contained predicted MHC binders nor did they align with any significance to any protein sequences in GenBank, suggesting that they were background noise. Contrastingly, as expected, evaluation of the recovered Ova minigene by NetMHCpan identified SIINFEKL as a strong MHC binder (IC50 = 44 nM) and BLASTP alignment to the GenBank database yielded OVAL (G*allus gallus)* as the top hit (E-score = 1.4 × 10^–18^). In the case of pmel-1 TCR/hgp100 screening, this analysis produced the expected spiked-in canonical hgp100 epitope, KVPRNQDWL, as a predicted MHC binder (IC50 = 123 nM) and aligned the detected spike-in hgp100 minigene to the reference human gp100 sequence (E-score = 3.0 × 10^–19^). However, in addition, we recovered a second minigene sequence encoding a peptide (KVYMPPIL) predicted to bind H-2Kb-encoded MHC with an IC50 of 409.9 nM, possibly representing a previously unreported epitope for this TCR.

To test whether the KVYMPPIL predicted peptide, or any other marginally enriched minigene sequence observed in the pmel-1 TCR/hgp100 screen, was a true antigen for the pmel-1 TCR, we re-screened the top 480 enriched sequences from Fig. 4a. The majority (313/480) of these sequences fell below the 10σ working cutoff used here, however, these minigenes were still included as it would still be possible for rare minigenes from the primary library to elicit T-cell reactivity, but not be detected as highly enriched due to low representation in the library. Synthetic oligonucleotide sequences were designed and generated by array-based synthesis. One consideration taken in the design of our panning library was the possibility that the 40 amino acid format hgp100 minigene that was used in the first round of screening may have had an enhanced propensity to be processed and presented on MHC compared with the 16 amino acid format minigenes that composed the random minigene library. To investigate this, we specified three different hgp100 minigenes, each of length 16 amino acids, to be included in the synthesized pool instead of the 40 amino acid version. Each of these short-version hgp100 minigenes were constructed such that the minimal KVPRNQDWL minimal epitope was placed in a different position of the minigene to also incorporate the possibility that position-specific effects could inhibit the immunogenicity of a given minigene.

The synthesized panning library was inserted into the FRET reporter-containing lentiviral transfer plasmid, and used to generate virus that was expressed in EL4 surrogate target cells and screened against pmel-1 TCR CTL. Amplicon sequencing performed on minigenes collected in FACS showed that the minigenes containing the KVPRNQDWL epitope were found to be the only minigenes from the set of 480 tested to meet the 10σ threshold in the second round (Fig. 4b).

In addition, from these results, we concluded that converting the 40 amino acid hgp100 minigene, which was used in initial library screening experiments, to a format mirroring the random minigene population did not appear to inhibit the ability of the minimal epitopes to be processed and presented by APC. To further confirm that this is the case, we constructed short-format versions of the Ova minigene and determined that there is no advantage bias with respect to %FRET-shift signal observed when long-format minigenes are co-cultured with OT-I CTL instead of the short format (Supplementary Fig. 7).

### Polyclonal T-cell populations can be used as input effectors

The validation experiments carried out using OT-I and pmel-1 transgenic mouse strains demonstrate feasibility when using large, monoclonal T-cell populations as input into minigene library screening experiments. However, a major practical consideration is the substantial initial effort that is necessary to isolate clonal T-cell populations-of-interest. Therefore, we tested our method with respect to a second definition of sensitivity by considering the limit of detection of T-cell reactivity, given a particular antigenic stimulus, when the reactive T cell is present in mixed T-cell populations in decreasing abundances.

As a first test, OT-I splenocytes, pmel-1 splenocytes, and wild-type C57BL/6 splenocytes were all activated and expanded by anti-CD3/28 stimulation and mixed together at varying ratios of model T cells (either OT-I or pmel-1 TCR CTL) to wild-type C57BL/6 CTL. These mixed OT-I and pmel-1 TCR CTL populations were then co-cultured with pure populations of EL4 cells expressing Ova minigene-FRET or hgp100 minigene-FRET cassettes, respectively, and assessed for their ability to elicit FRET-shift signal. In the case of both the OT-I CTL and the pmel-1 TCR CTL, it was observed, as expected, that as the abundance of minigene-reactive model CTL decreased, the proportion of cells undergoing FRET-shift also decreased. However, in both cases, we found that even at 1:3000 abundance, the model TCR CTL were able to result in statistically significant FRET-shift signal relative to wild-type C57BL/6 splenocyte-derived CTL alone (Supplementary Fig. 8).

**Fig. 5:**
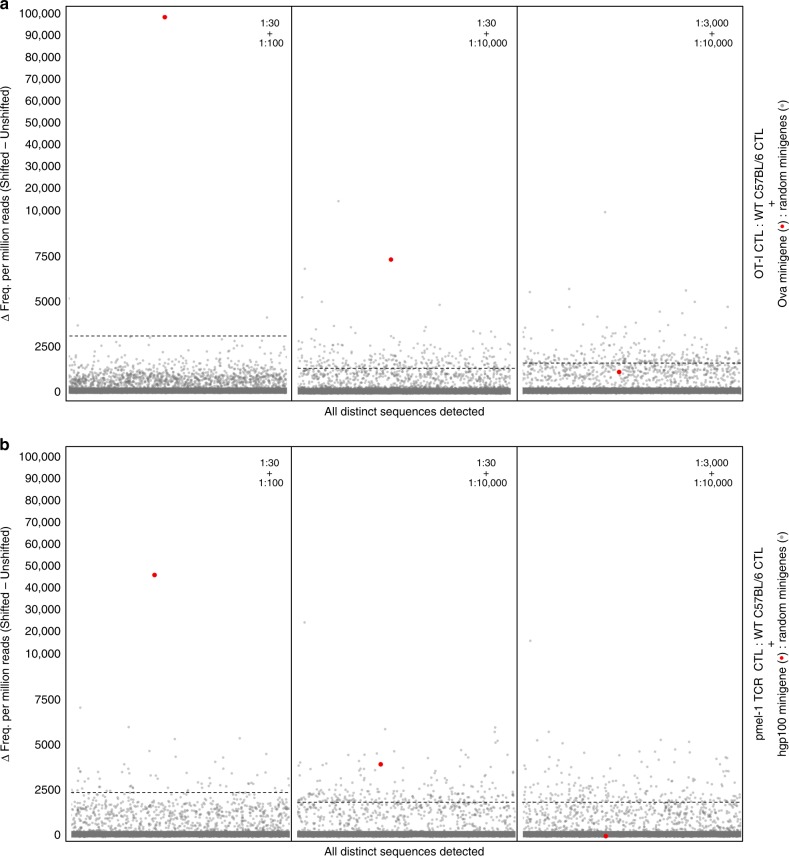
Screening co-cultures of mixed CTL populations + mixed target cell populations. Random minigene cell libraries spiked with either Ova minigene-expressing cells or hgp100 minigene-expressing cells were co-incubated with wild-type C57BL/6 CTL spiked with OT-I or pmel-1 TCR CTL, respectively. The specific mixtures used for testing are described in panel headers. Target and CTL populations were combined at a 1:1 ratio and co-cultured for 4 h before being sorted on FRET-shift status. Recovered cells from Shifted and Unshifted gates from each screening condition were lysed, and integrated minigenes were amplified from genomic DNA using PCR primers specific for the conserved transgene region flanking the minigene site. Illumina adapters and indexes were added in a second round of PCR, and resultant amplicons were sequenced on the Illumina MiSeq platform with 2 × 250 paired-end chemistry. **a** The differences in relative abundance for all distinct minigene sequences detected in the Shifted gates and the Unshifted gates of OT-I/Ova minigene mixtures. **b** The differences in relative abundance for all distinct minigene sequences detected in the Shifted gates and the Unshifted gates of pmel-1 TCR/hgp100 minigene mixtures

Next, we tested epitope detection using mixed populations of target cells screened against mixed populations of CTL. Three separate “mixed + mixed” co-culture conditions were prepared for each model TCR/antigen pair, screened on FRET-shift status, and subject to minigene amplicon sequencing. Raw reads were processed and analyzed as in the previous experiments, and we found that, for both model TCR/antigen pairs, canonical minigenes were enriched 10σ above background out of a 1:10,000 mixture when T cells were diluted to an abundance of 1:30 (Fig. 5).

Extending these investigations, we then tested the ability of our assay to detect antigen from a library when polyclonal T-cell populations from a biological context are screened and sequenced, rather than prepared populations of transgenic T cells and wild-type T cells. To this end, we engrafted wild-type C57BL/6 mice with either B16F10 tumor cells stably expressing full-length Ova protein (B16F10-Ova) or parental B16F10 cells with no Ova. After 16 days of tumor growth, mice carrying B16F10-Ova tumors were either vaccinated with Ova protein^41^ to simulate conditions of an active antitumor response (Supplementary Fig. 9) or left unvaccinated. From all mice, we subsequently isolated tumor tissue on day 21, performed FACS to isolate CD8^+^ tumor-infiltrating lymphocytes (TIL), and activated/expanded them by anti-CD3/28 stimulation. From both the parental B16F10 tumor mice and B16F10-Ova mice with no vaccine boost, we were unable to expand a sufficient number of viable CD8^+^ cells for use in screening, indicating that these mice were not mounting a detectable T-cell response to tumor antigen.

**Fig. 6:**
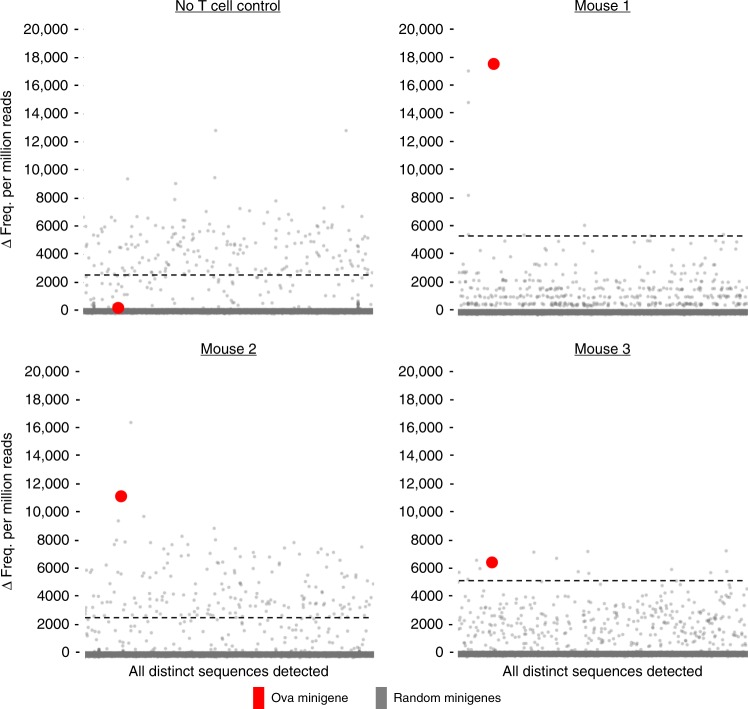
FRET-shift/amplicon sequencing using tumor-infiltrating T cells as input effectors. B16F10-Ova tumor masses were engrafted into wild-type C57BL/6 mice and boosted by vaccination with Ova prior to isolation of tumor-infiltrating CD8^+^ T cells by FACS, further expanded by stimulation with plate-bound anti-CD3/28, and co-cultured with random minigene library cells spiked with Ova minigene-expressing cells at 1:10,000 abundance. Three replicate mice were prepared, and TIL samples from each were independently screened against the spiked library cell population. Limited outgrowth ex vivo of the TIL populations necessitated that co-cultures be conducted at reduced E:T ratios, ranging from 0.5:1 to 0.7:1 across the replicates (4 h length). In parallel to TIL samples, a matched negative control sample was prepared by performing FRET-shift FACS/amplicon sequencing on spiked library target cells that were not subjected to co-culture with any T cells. The Δ relative abundance of all detected minigenes are displayed for the three replicate TIL screens as well as the no-T-cell control. The dashed line represents 10σ above the mean Δ relative abundance value

We were, however, able to isolate and expand TIL from B16F10-Ova mice that had received Ova vaccination. By tetramer staining, we observed that the obtained TIL were between 1.77% and 5.30% H-2kb/SIINFEKL tetramer positive (mean = 3.68% across the three mice) (Supplementary Fig. 10a). Upon anti-CD3/28 stimulation and expansion of the cells in the presence of IL-2, the resultant T cells were co-cultured with 1 in 10,000 mixtures of Ova-spiked random minigene-expressing EL4 cells. Co-cultured target cells were sorted on FRET-shift status (Supplementary Fig. 10b, c) and subjected to minigene amplicon sequencing. The results indicate that in all three TIL samples, derived from three replicate mice, the Ova minigene was, again, readily detectable above the 10σ cutoff while no enrichment of the Ova minigene was detected in the no-CTL control (Fig. 6).

## Discussion

In the work presented here, we have demonstrated a FRET-shift/amplicon-sequencing approach to simultaneously assess vastly more epitopes for their ability to be processed, presented, recognized, and elicit reactivity than would be tractable with conventional methods^42^. The experiments here show that, even when present at frequencies as low as 1 in 10,000, relevant antigen can readily be detected above background. However, it is possible that for scaled-up versions of these studies in which naive library screening with no spiked-in test antigen are performed, it should be attainable to screen 1 × 10^6^ minigene sequences for T-cell reactivity.

This estimate is based on the postulate that, given the 1:1 effector:target ratio and the small scale of 3 × 10^6^ target cells per condition used in the experiments performed here, a copy number of 300 cells carrying a given antigenic minigene is approximately the minimum threshold number that is needed for detection by FACS and amplicon sequencing (1:10,000 = 300 cells/3,000,000 cells). For naive library screening with no a priori knowledge of antigens composing the library, it would be necessary to meet this level of clonal redundancy for all distinct minigene-expressing clones in the population. To achieve this with 1 × 10^6^ minigene sequences, it then follows that co-culture experiments should be done at a scale of roughly 300 million target cells per screen, which is a practically achievable parameter.

We have also shown that this strategy is sufficiently robust to detect individual T-cell reactivities out of polyclonal mixtures of T cells when their abundance is ~1–5%. Though other methods currently in use today show superior performance in terms of detecting very low abundance T-cell clonotypes in complex T-cell repertoires, the ability of the FRET-shift/amplicon-sequencing method should still be useful for screening T-cell populations taken from relevant biological contexts. T cells taken directly from disease lesions or the peripheral blood of vaccinated individuals are occasionally observed to contain antigen-specific T cells present at > 1% frequency^43,44^. However, when such T-cell frequencies of antigen-specific T cells are not met, sophisticated strategies to rationally enrich for reactive T cells by activating sub-pools of PBMC have been used to generate “mini-lines” in which reactive T cells are known to be present at a minimum threshold frequency^45,46^.

**Fig. 7:**
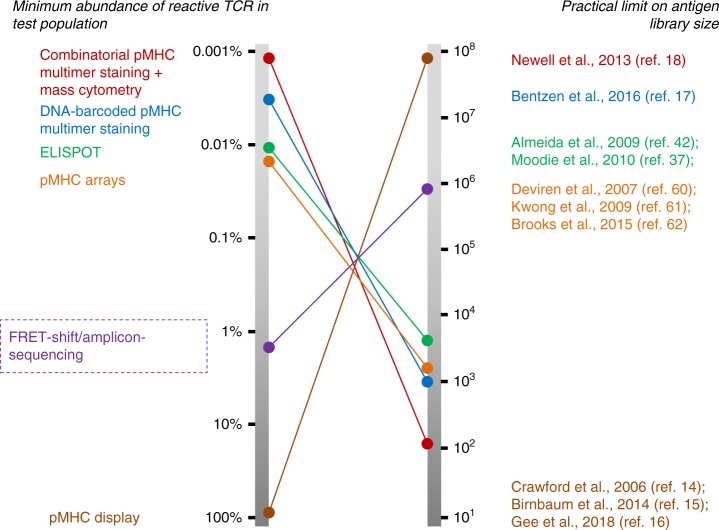
Comparison of our strategy with methods currently in use for T-cell antigen discovery. The methods currently constituting the suite of approaches available currently for T-cell antigen screening as well as our FRET-shift/amplicon-sequencing methodology are mapped with respect to their robustness in detecting T-cell antigens from highly mixed T-cell populations (left) and their ability to screen high-diversity libraries of candidate antigens (right)^14–18,37,42,60–62^. It will be possible to place the recent methods described by Li et al.^47^, Joglekar et al.^48^, and Kisielow et al.^49^ on these spectra when data are published showing method sensitivity with respect to polyclonality of input T-cell populations and complexity of input antigen libraries

Various methods available to perform T-cell antigen screening is illustrated in Fig. 7. The FRET-shift/amplicon-sequencing approach validated here is conceptually oriented in a direction opposite to most current methods, which were originally developed from the perspective of profiling the reactivity of repertoires of T cells against small panels of antigens-of-interest. In contrast, we propose our approach as a means to assess T-cell clonotypes which have already been determined to be interesting or relevant and, by exposing them to large libraries of candidate antigens, understanding the repertoire of epitopes that they recognize.

Other methods have recently been developed to conduct high-throughput T-cell antigen discovery using this rationale of isolating putative antigen-expressing target cells from bulk library populations, via a target cell-based read-out, and then characterizing recovered cells by next-generation sequencing analysis. In these, the use of either single-chain peptide-MHC molecules^47^ or chimeric MHC-CD3ζ molecules in conjunction with an NFAT-linked reporter^48,49^ is required. In the former format, these single-chain pMHC configurations are used to generate supraphysiological numbers of pMHC complexes at the APC surface and, in so doing, bypass the natural antigen-processing and presentation pathway and run the risk of identifying non-naturally occurring peptide epitopes. In the latter configuration, MHC-CD3ζ chimeras are used to approximate signaling through the native TCR complex and infer activation. However, this design alters the endogenous immune synapse, which is known to be critical to normal T-cell activation by balancing pro-signal cascade kinase activity and signal-suppressing phosphatases^21^ or by modulating the electrostatic interactions between TCR complex ITAM domains and the inner leaflet of the T-cell membrane during physiological T-cell activation^50^. In contrast, the FRET-shift/amplicon-sequencing approach described here leverages the natural antigen processing and presentation pathway operating in target cell populations, maintains the biophysical context of natural TCR/pMHC receptor–ligand interactions, and monitors the function of activated cytotoxic T cells.

In other high-throughput T-cell antigen profiling efforts^8,15,16^, libraries of up to 10^8^ distinct minigene sequences have been constructed and screened with T cells or TCR-based soluble reagents. However, it is important to consider that these methods rely on plasmid transfection of APC. Transfection results in the delivery of a very high multiplicity of minigene plasmids to host cells^51,52^ and, therefore, these approaches require a number of rounds of iterative screening (panning) in order to sufficiently enrich the signal for identification. In contrast, the FRET-shift/amplicon-sequencing assay, while only querying on the order of 10^6^ unique minigenes, did not require multiple rounds of panning to identify antigenic minigenes. In other words, the reduction taken in library diversity caused by lentiviral transduction comes with the dividend that antigens could be identified immediately with relatively little post hoc deconvolution.

For the studies, we present here, mouse T cells were used to perform proof-of-principle experiments because of the availability of MHC-matched APC lines and sources of clonal T populations from TCR-transgenic models. These materials allowed for iterative development and testing of the FRET-shift FACS/deep amplicon-sequencing approach with respect to method kinetics, specificity, sensitivity, and robustness. However, these methods are not restricted to mouse systems and are readily portable to human cells. The GZMB-cleavable amino acid sequence used here is known to also be specifically acted upon by human GZMB^32,33^, indicating that the FRET reporter used herein is usable in human experiments without modification. The main challenge associated with applying high-throughput antigen screening to the study of T-cell populations from highly outbred human donors requires more advanced strategies for ensuring histocompatibility between target and T cells used in library screening. In this regard, K562-based artificial APC have been developed in recent years, and have been validated as a reliable means to generate MHC-matched APC with intact antigen processing and presentation^53^.

Indeed, recent work has been done that builds on the extensive benchmarking of the methodology that we describe here. The application of GZMB-based T-cell antigen screening to microbial and human reference proteomes in the context of recombinant human TCR in primary human CTL^54^ provides an additional validation of the core concept that we have described previously^55^. Looking ahead, we anticipate that the method we report here will prove useful in surveying unbiased libraries of peptide-coding sequences to provide a more complete view of T-cell receptor/epitope interactions and, thereby, allow researchers to better understand basic T-cell biology, develop better predictive models of T-cell reactivity, and rationally design T cell-based immunotherapeutics for the treatment of cancer, infectious disease, transplant rejection, and autoimmune disorders.

## Methods

### Animal ethics statement

All animal experiments were assessed and approved by the University of British Columbia’s Animal Care Committee under ethics certificate #A18-0197.

### Cell culture

All cell cultures were maintained in the RPMI^−^1640 supplemented with 2 mM GlutaMAX, 1 mM sodium pyruvate, 50 μM β-mercaptoethanol, 10 mM HEPES, 100 U/mL penicillin, 100 U/mL streptomycin, and 10% heat-inactivated fetal bovine serum. Culture media and supplements were all sourced from Gibco. Cultures were maintained at 37 °C and 5% CO_2_ atmosphere.

### CTL activation and expansion

Fresh-frozen dissociated splenocytes from OT-I mice (a gift of Dr. B. Nelson, BC Cancer Agency Deeley Research Centre), pmel-1 TCR mice (from intact spleens shipped by The Jackson Laboratory), or wild-type C57BL/6 mice were thawed, washed, adjusted to a density of 5 × 10^6^ cells/mL in supplemented RPMI + 50 U/mL rhIL-2 (Peprotech). Cells were seeded into a flat-bottom 96-well plate (1 × 10^6^ cells/well) that had been pre-coated overnight with low endotoxin, azide-free anti-mouse CD3 (clone 145-2C11), and anti-mouse CD28 (clone 37.51) (Biolegend) and washed prior to addition of cells. Cells were removed from coated plates after 48 h, adjusted to 1 × 10^6^/mL by adding fresh media + rhIL-2 to conditioned media, and cultured in U-bottom 96-well plate wells. Expanding T cells were split again on day 5, adjusting density to 1 × 10^6^, and used in experiments on day 7.

### Construction of vector and minigene inserts

The lentiviral transfer plasmid was derived from the pCCL-c-MNDU3-PGK-EGFP backbone. The PGK-EGFP portion of the plasmid was replaced with a custom multiple-cloning site via the EcoRI/BamHI sites in the original vector (which were ablated during ligation). Enhanced YFP and mCerulean (CFP)-coding sequences were PCR amplified from the Addgene plasmids #11180 and #15214 using Phusion High-Fidelity polymerase (New England Biolabs). The CFP forward primer was tailed with P2A-coding sequence, while the YFP forward primer was tailed with the GZMB-cleavable substrate-coding sequence. Tailed CFP and YFP amplicons were then sequentially inserted into the lentiviral transfer vector backbone pCCL-c-MNDU3-MCS intermediate using FseI/AgeI and AgeI/AscI restriction cloning, respectively. A stuffer fragment ~1 kb in length containing multiple in-frame stop codons was placed upstream of the P2A sequence via BamHI/EcoRI restriction cloning to yield a parental minigene acceptor plasmid, referred to as pMND-silent-FRET, that remains fluorescently silent until the stuffer is swapped for productive minigenes. The Ova-minigene was prepared by amplifying directly from cDNA recovered from the ID8.G7 cell line, which stably expresses full-length ovalbumin protein. Ova scrambled control and hgp100 minigene inserts were prepared by overlap extension PCR of ssDNA oligos (Supplementary Table 4). Ova, Ova-scrambled, and hgp100 minigenes were cloned into linearized pMND-silent-FRET parental backbone via BamHI/EcoRI restriction cloning. Double-stranded random minigenes were generated by performing 30 cycles of PCR on a synthesized RM_template ssDNA (20 nM final concentration) with RM_FWD and RM_REV (Supplementary Table 4). Random minigene libraries were BamHI/EcoRI digested, purified by 3% agarose gel electrophoresis, and inserted into pMND-silent-FRET. All above oligonucleotide syntheses were performed by Integrated DNA Technologies. Panning library for second-round screening of pmel-1 TCR was synthesized as an array-based oligonucleotide pool by CustomArray. Minigene flanking regions were designed exactly as done for random minigene library oligos, and subsequent pmel-1 TCR panning library was constructed as described above for random minigene library construction.

### Virus production

To generate Ova, Ova-scrambled, or hgp100 minigene lentivirus, 40 µg of each transfer plasmid was separately combined with 36 µg of pCMV-ΔR8.91 and 4 µg of pCMV-VSV-G plasmids. These DNA mixes were incubated with 7.5 mL OptiMEM (Gibco) and 0.5 mL of TransIT-LT1 reagent (Mirus) for 30 min at room temperature. To 8 × 10 cm culture plates containing 40% confluent HEK293T cells, 1 mL of transfection mix was added per plate for each minigene. To generate random minigene lentivirus, 80 µg of transfer plasmid was combined with 72 µg of pCMV-ΔR8.91 and 8 µg of pCMV-VSV-G plasmids. These DNA mixes were incubated with 15 mL OptiMEM (Gibco) and 1 mL of TransIT-LT1 reagent (Mirus) for 30 min at room temperature. To 16 × 10 cm culture plates containing 40% confluent HEK293T cells, 1 mL of transfection mix was added per plate. In all cases, media was replaced 18 h post transfection, and viral supernatants were then collected at 36, 48, 60, and 72 h post transfection. To concentrate virus, supernatants were ultracentrifuged (110,000 RCF, 90 min, 4 °C), and pellets were resuspended in 1 mL OptiMEM (Gibco). Titers of viruses were determined by testing (in duplicate) 1, 2, 4, 8, 16, or 32 μL of concentrated virus on 5 × 10^4^ HeLa cells in 24-well format with a final volume of 500 μL of complete culture media. Transduction efficiency was determined by measuring the % of fluorescent cells detected in flow cytometry.

### Viral transduction

For transduction of ID8 cells with Ova or Ova-scrambled minigene viruses, 4 × 10^6^ cells were plated at ~50% confluency in a 1 × 10 cm tissue culture dish per virus. Concentrated viruses (0.4 mL) were each diluted with 2.6 mL of complete media and each added to plates. Plates were incubated in 3 mL volumes for 18 h, 37 °C, at which point cultures normal culture was resumed in 10 mL of complete media. For transduction of random minigene virus, 1.2 × 10^7^ ID8 cells were plated at ~50% confluency in 3 × 10 cm tissue culture dishes. Concentrated virus (1.2 mL) was diluted with 7.8 mL of complete media, and 3 mL of transduction mix was added to each plate. Plates were incubated in 3 mL volumes for 18 h, 37 °C, at which point normal culture was resumed in 10 mL of complete media. For transduction of EL4 cells with Ova or hgp100 minigene viruses, 20 μL of concentrated virus was added to 1 × 10^5^ cells in 500 μL of complete media in a single well of a 24-well plate. After 24 h, the cells were diluted to 5 mL in complete media and transferred to a T-25 flask for continuous culture. For transduction of random minigene virus, EL4 cells were adjusted, in complete media, to a density of 7.5 × 10^5^ cells/mL in a final volume of 150 mL. To these cells, 1.6 mL of concentrated viral stock was added and the resulting cell/virus mix was split across 15 × 10 mm dishes (10 mL per dish).

### Mouse tumor grafts

Four- to five-week-old male C57BL/6 mice from the BC Cancer Research Centre’s Animal Resource Centre, maintained under pathogen-free conditions, were engrafted with 3 × 10^5^ B16F10 cells ( + /− Ova transgene) by subcutaneous injection in the scruff of the neck. Groups receiving Ova vaccination were also subcutaneously injected with 300 μL sterile-filtered PBS solution containing chicken egg ovalbumin protein (MilliporeSigma) (100 µg) and the TLR3 adjuvant, poly(I:C) (InvivoGen) (10 µg) on day 16 to day 19 post initial tumor graft at ~24 h intervals. Mice were euthanized on day 21, and the tumor masses were removed, dissociated, and subject to FACS (using single-cell level purity masking) to obtain pure populations of CD8^+^ TIL. Cells were in vitro stimulated and expanded in mixed lymphocyte reactions with irradiated (50 Gy) feeder C57BL/6 splenocytes at a 100:1 feeder: TIL ratio. Stimulations were performed in single wells of 24-well plate containing 500 µL of supplemented RPMI + 100 ng/mL each of anti-CD3/28 mAb (same clones as above) and 400 IU/mL rhIL-2 (Stemcell Technologies). Media + rhIL-2 was completely replaced on day 3, and cells were continued in culture for a further 15 days, replacing half of conditioned media with fresh media + rhIL-2 every 3 days, prior to their use in experiments.

### CTL/APC co-cultures

Activated and expanded CTL were used in FRET-shift assays on 7 days post stimulation. Cell populations were enumerated using the Countess automated cell counter (Invitrogen) to determine input cell number for CTL/APC co-cultures. For FRET-shift assays using ID8-derived target cells, co-cultures were performed for 4 h at a 1:1 effector:target ratio in round-bottom 12 × 75 mm FACS tubes containing 1 × 10^5^ target cells. For random minigene-expressing ID8 library screening, co-culture size was scaled up to 8 × 10^5^ target cells/tube, and four tubes were prepared per screening condition. Upon completion co-cultures, replicate tubes were pooled prior to FACS analysis. For FRET-shift assays using EL4-derived target cells, co-cultures were performed for 4 h at a 1:1 effector: target ratio in U-bottom 96-well plates containing 1 × 10^5^ target cells/well. For random minigene-expressing EL4 library screening, 32 wells per screen were prepared and pooled prior to FACS analysis. In all cases, co-cultures were maintained at 37 °C, in 5% CO_2_ atmosphere.

### Flow cytometry/FACS

Virally transduced APC lines were purity-sorted to remove untransduced targets and cells carrying non-productive minigene-reporter transgenes. A representative screenshot of the FRET purity-sorting gating template is shown in Supplementary Information (Supplementary Fig. 11). Cells were either sorted on BD FACSAria Fusion or BD FACSAria II by gating for single-cells (as determined by FSC-A vs. FSC-W and SSC-H vs. SSC-W) that were PI^−^ (ex. 561, em. 610), YFP^+^ (ex. 488, em. 530) and emitting resting FRET signature in FRET (ex. 405, em. 525/50 + 505LP) vs. CFP (ex. 405, em. 450/50) plots. For analysis of CTL co-cultures, cells were washed 1X with PBS, resuspended in 500 μL PBS + 5% FBS + 1 μg/mL propidium iodide (PI), filtered through 40 um nylon mesh, and kept on ice for duration of analysis. Cytometric analyses were performed on BD LSR II Fortessa, and FACS cell isolation was done on BD FACSAria Fusion. A representative screenshot of the FRET-shift FACS gating template is shown in Supplementary Information (Supplementary Fig. 12). Target cells to be analyzed were selected by gating for PI^−^ and YFP^+^ singletons. Cells undergoing T-cell targeting were sorted on the basis of FRET-shift in FRET (ex. 405, em. 525/50 + 505LP) vs. CFP (ex. 405, em. 450/50) plots. Gated cells were collected in 15 mL conical Falcon tubes containing 3 mL of 55% FBS/45% culture medium. Immediately after collection, cells were pelleted at 300×*g*, 5 min and subjected to gDNA isolation using DNAzol reagent (Invitrogen).

### Quantitative PCR

Copies of Ova minigene and scrambled minigene in the genomic DNA of collected cells from mixed target populations were enumerated using a custom duplex TaqMan assay (Applied Biosystems; assayID: AHUAOFV; TaqMan Universal PCR Master Mix) able to discriminate between Ova minigene and scrambled minigene. A 5-point standard curve ranging from 100,000 copies to 100 copies of purified plasmid containing target sequences for either minigene was constructed. Unmixed Ova minigene-expressing cells, and scrambled minigene-expressing cells were included as controls to correct for differences in average insert copy numbers between the two populations. Samples were performed in sets of four technical replicates.

### Sequencing

Sequencing libraries were generated by amplifying minigenes from isolated gDNA of gated cells. Primers were designed to anneal to regions flanking the minigene site on the viral transgene (Supplementary Table 4) and were either directly tailed with Illumina adapterized primers containing population-specific index sequences during oligo synthesis or tailed with Illumina adapters/indexes during a second round of PCR. Sequencing libraries for Fig. 3 experiment were prepared by single-round PCR: 25 cycles were performed with Phusion polymerase (New England Biolabs) and indexed amplicons were pooled and gel-purified. All other sequencing libraries were prepared using a two-round PCR protocol in which 21–30 cycles were run in first round, 50% of first-round product was carried forward for second round, and 10 cycles were run in second round PCR (both using Phusion polymerase) prior to pooling and gel-purification. In all cases, sequencing was performed on the Illumina MiSeq platform using PE250 chemistry. To improve %PF during cluster-formation steps, PhiX control DNA was added to flow cells, and/or a staggered primer strategy was employed to ensure sufficiently even base-pair distribution at each cycle. Upon completion of sequencing runs, paired-end reads were assembled to form minigene-contigs using FLASh^56^ with default parameters except for max-overlap = 304 and “allow outies” functionality enabled. Assembled reads were quality filtered (keeping reads with 90% of bases > Q20) and trimmed using FASTX-Toolkit^57^. Reads were then clustered using Starcode^58^ on default parameters to collapse divergent sequences arising from PCR or sequencing error. Any sequence clusters containing residual stop codons that were not effectively filtered out by purity sorting were removed, and further manual inspection and curation of recovered minigenes was then applied to remove prevalent known PCR or in silico artifacts from data sets prior to further analysis. For prediction of MHC binders from detected minigenes, NetMHCpan 3.0 was used with default parameters. For database searching of detected minigenes, BLASTP alignment to all nonredundant GenBank CDS translations + PDB + SwissProt + PIR + PRF excluding environmental samples from WGS projects (release 218.0)^59^ was performed using default parameters (with automatic adjustment for short input sequences).

### Data analysis

Gel and CFU analyses were performed using ImageQuant TL v8.1. Flow-cytometry analyses were performed using FlowJo V10.0.8r1. Quantitative PCR analyses were performed using SDS2.4. Sequence data handling was performed using Geneious v8.1.2 and Bioconductor package seqinr. Other data handling and statistical analyses were performed using R v3.1.2 and RStudio.

### Reporting summary

Further information on research design is available in the Nature Research Reporting Summary linked to this article.

## Supplementary information

Supplementary Information

Peer Review file

Reporting Summary

Source Data

## Data Availability

The authors declare that the DNA sequencing data that support the findings of this study are available as raw merged paired-end read FASTQ files in the Zenodo repository with the identifier 10.5281/zenodo.3385274. Other data types that support the findings of this study are available from the corresponding author upon request.

## Code availability

Custom code used in this study is available from the corresponding author upon request.
