# Supplementary material for: Rapid selection and identification of functional CD8+ T cell epitopes from large peptide-coding libraries
Source: Nat Commun. 2019 Oct 7;10:4553. doi: 10.1038/s41467-019-12444-7 (PMC6779888; doi:10.1038/s41467-019-12444-7)
Supplement: Supplementary file 1 — Supplementary Information [file 41467_2019_12444_MOESM1_ESM.pdf]

Rapid selection and identification of functional CD8+ T cell epitopes from large peptide-coding libraries

Sharma et al.

Supplementary Information

Supplementary Figures 1-12
Supplementary Tables 1-4

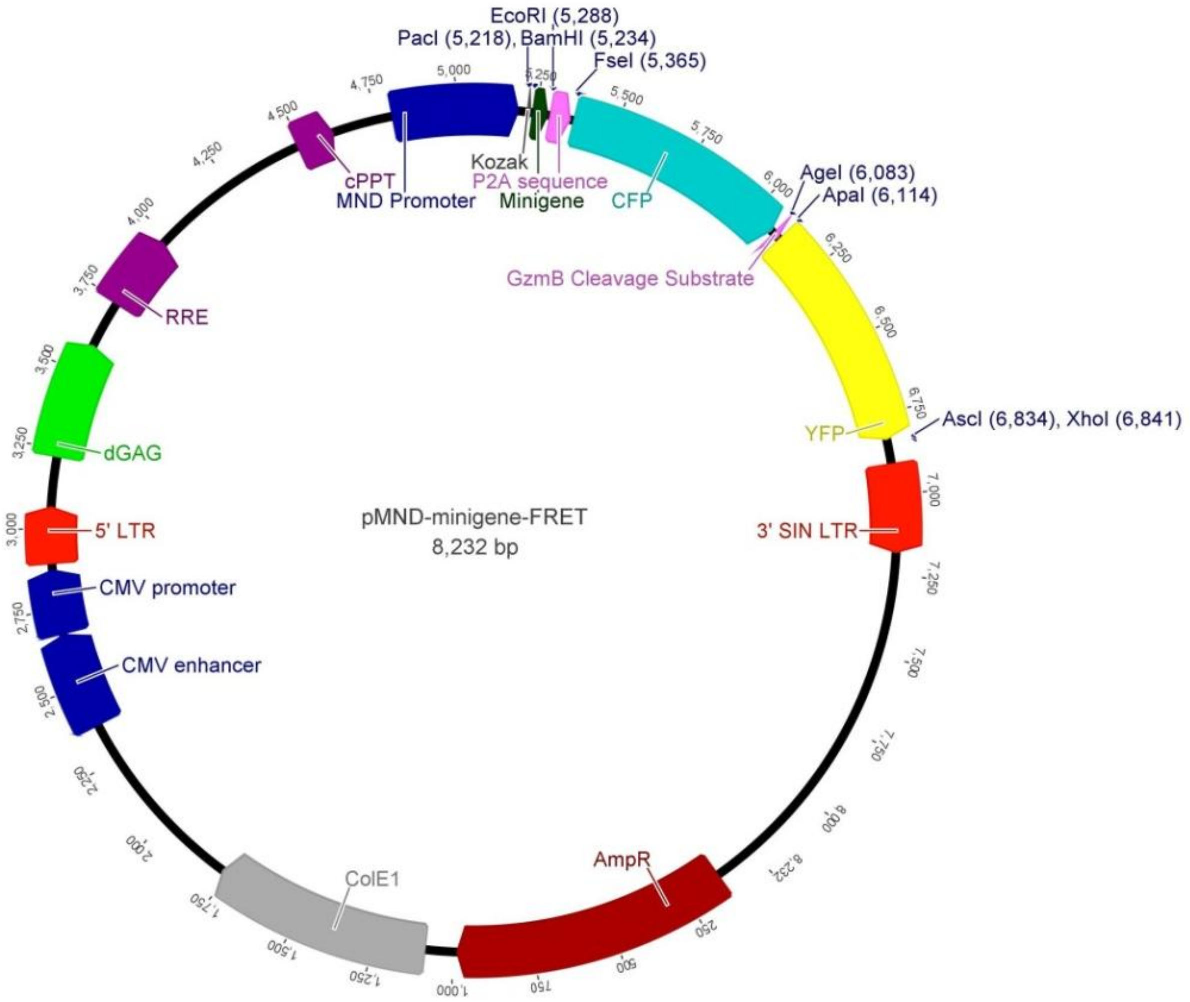

Supplementary Figure 1. General-purpose plasmid map of lentiviral transfer vector. Fully annotated plasmid map of minigene-FRET lentiviral transfer plasmids used in these experiments.

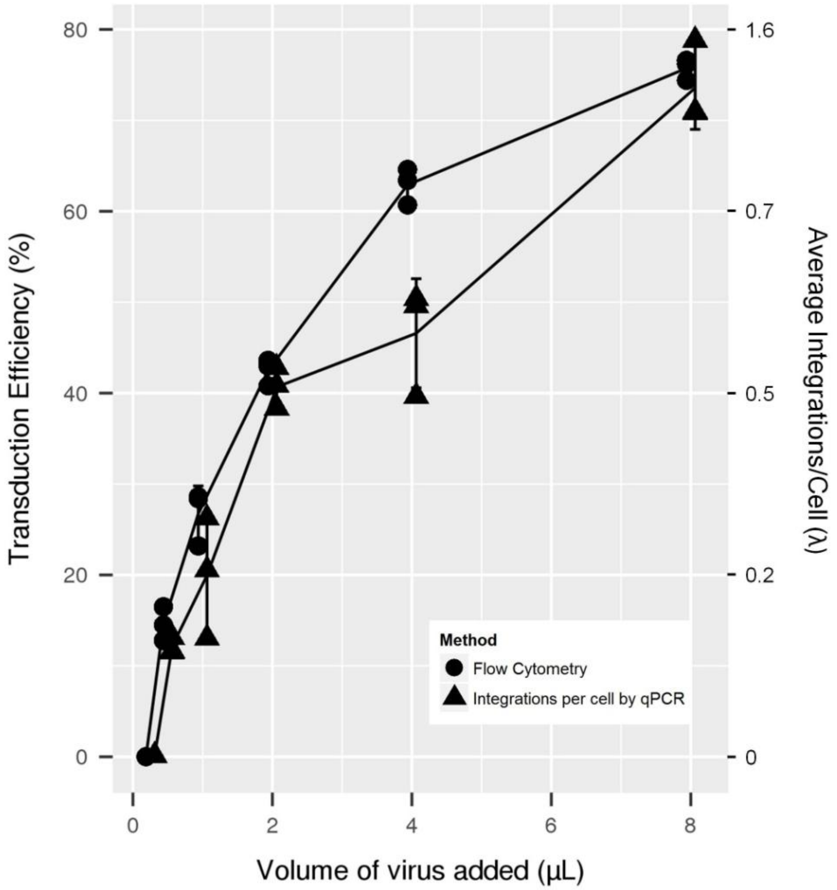

Supplementary Figure 2. Validation of the in-house integrations/cell qPCR assay. A qPCR assay employing SYBR Green reagent and an MND promoter-specific primer set (Suppl. Tab. 2) in parallel with TaqMan reagent and a mouse beta actin 3'UTR-specific TaqMan primer/probe set (ABI part #4352933E) for analysis of transduced cells at the genomic level. Parallel standard curves constructed from a plasmid containing MND and mActB target sequences are analyzed by each primer set to infer the number of lentiviral integrations (using MND read-out) and total cell number (using actin read-out) present in each sample. The average integrations/cell can then be calculated from these values. To benchmark the assay, a virus titering curve was prepared and the proportion of transduced cells was measured by flow cytometry. Integrations/cell were measured on the same cell populations by qPCR and used to calculate the proportion of cells carrying at least one integrated viral cassette using the equation, $P(k > 0) = 1 - e^{-\lambda}$. The resulting data from both methods (three biological replicates per sample, error bars denote SD) were tested with a two-sample Kolmogorov-Smirnov test at 95% confidence and found to be not statistically different. Since Poisson-based estimation of viral integrations cannot be estimated by proportions of fluorescent cells in transduced populations that have been sorted to purity, validation of the qPCR-based assay provides an avenue by which the average number of viral integrations per cell can be measured in target populations prior to their use in screening assays.

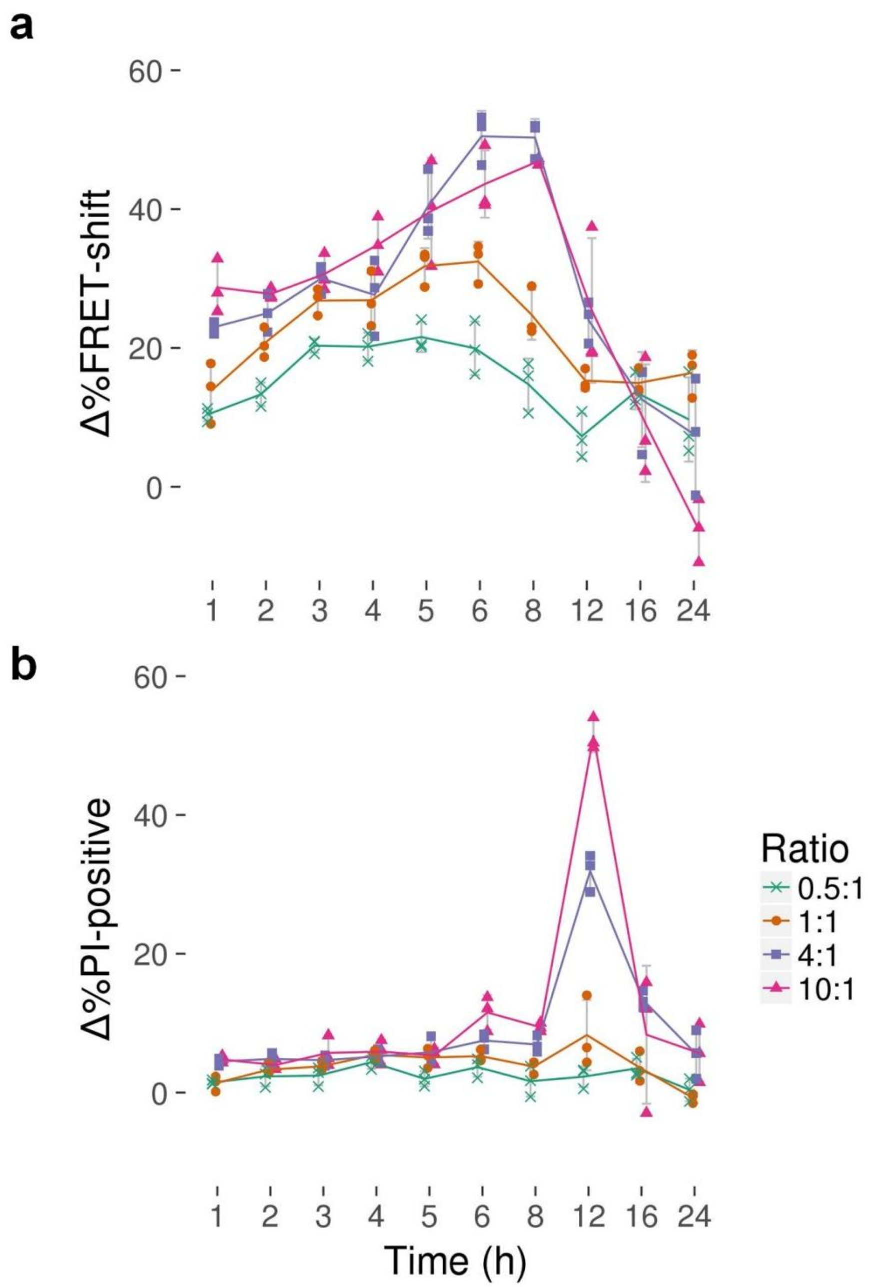

Supplementary Figure 3. OT-I time-course. Ova minigene-expressing target cells were exposed to varying lengths of time with OT-I T cells at different effector: target ratios. The proportions of cells undergoing (a) FRET-shift and (b) entering apoptosis (as measured by PI uptake) were both monitored for each time-point and ratio. All individual data points are shown as values standardized to the mean of the CTL- control at each time point; illustrated lines pass through group means, error bars denote SD.

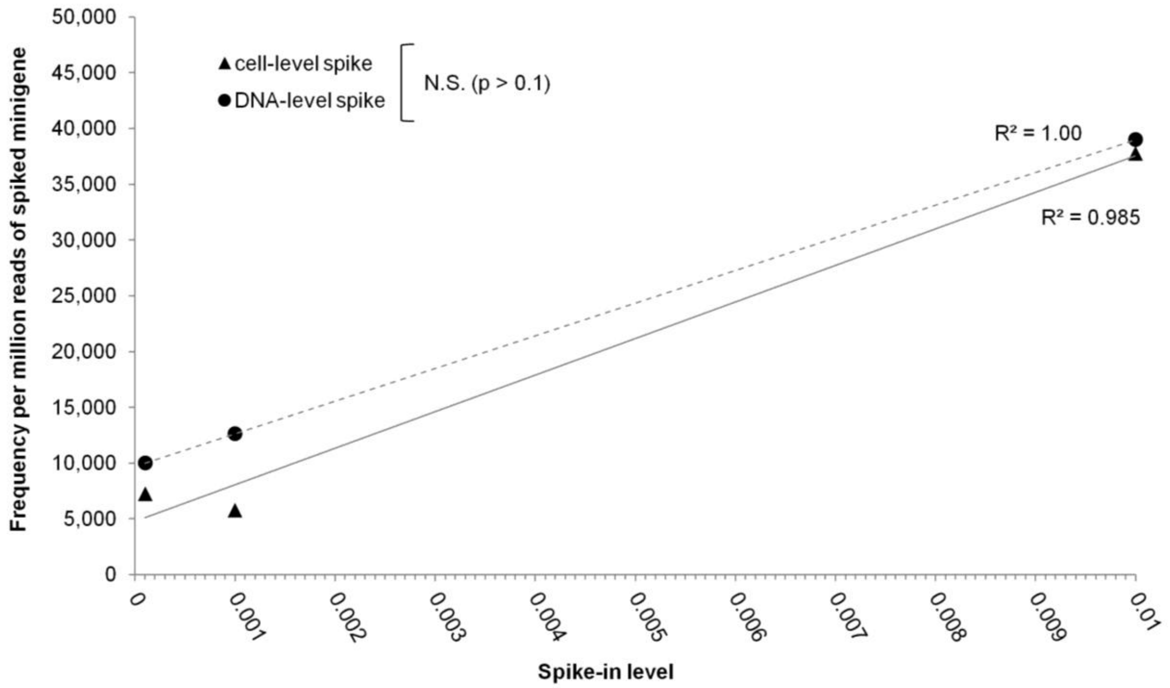

Supplementary Figure 4. Comparison of minigene spike-in methods used in this study. Ova-spiked random minigene library cells were produced by either mixing Ova minigene-containing lentiviral transfer plasmid with random minigene-containing plasmid prior to generating virus and transducing EL4 cells, or mixing together Ova minigene-expressing EL4 cells and random minigene-containing EL4 cells. Ratios of 1:100, 1:1,000, and 1:10,000 were prepared by both methods. Amplicon sequencing was performed on minigenes recovered from all prepared populations and comparison of the read frequencies obtained by multiple linear regression indicated that the choice of spike-in method did not result in a statistically significant difference between the two ($p > 0.1$).

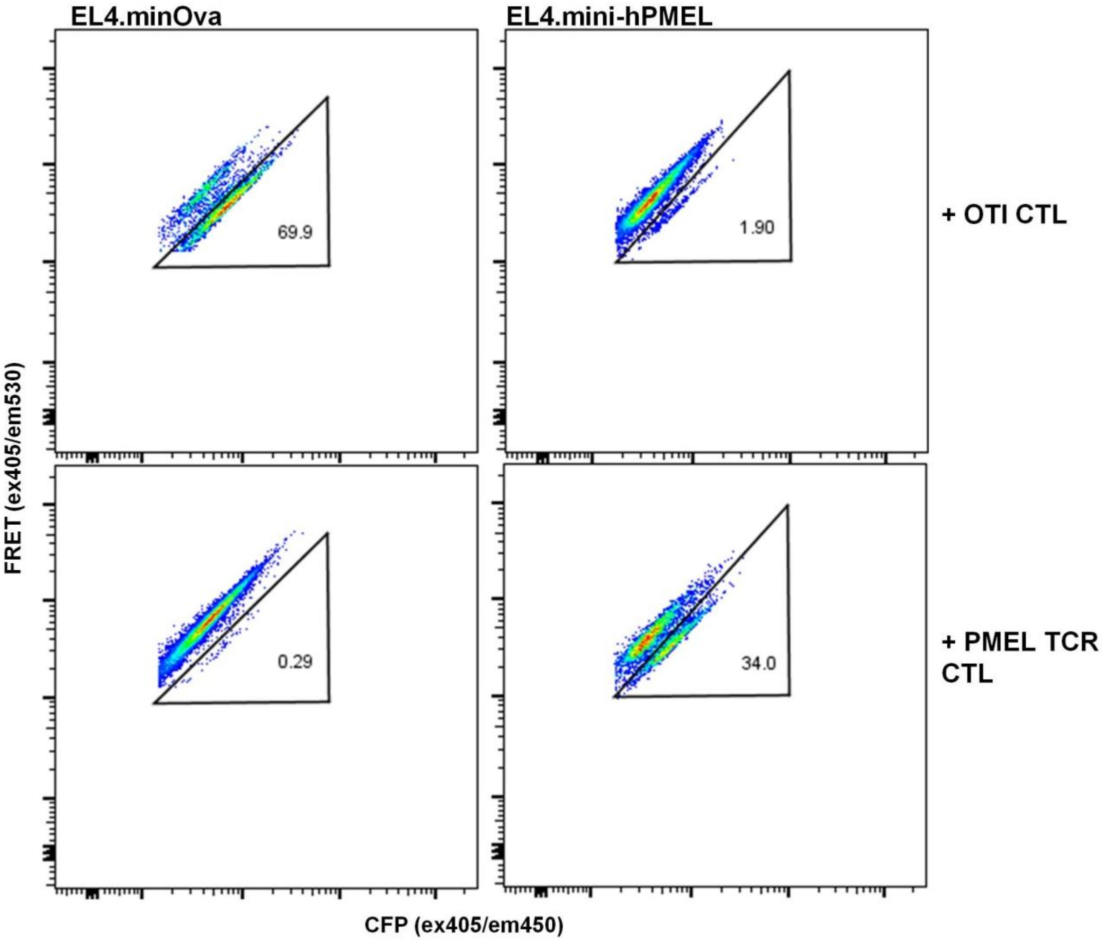

Supplementary Figure 5. The size and magnitude of FRET-shift is influenced by the identity of the target cell. EL4 cells were transduced with Ova and hgp100 minigene viruses. When co-incubated with either expanded OT-I CTL or pmel-1 TCR CTL, an antigen-specific FRET-shift signal is developed. The size and magnitude of the observed FRET-shift in Ova-expressing EL4 cells is greater than that previously observed in ID8 cells.

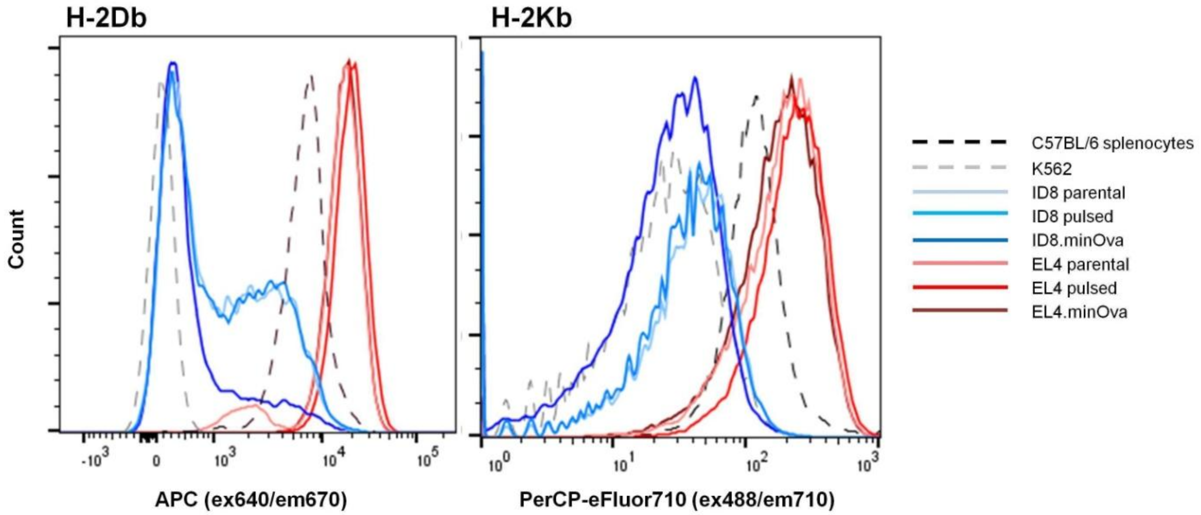

Supplementary Figure 6. Host target cell lines vary significantly in MHC-I expression. EL4 cells express both the known C57BL/6 mouse MHC-I alleles, H-2Kb and H-2Db, at much higher levels than do ID8 cells. No differences were observed in the H-2 expression in either cell type between peptide-pulsed, minigene-transduced, or parental cells. K562 cells (human cell line) were included as a negative control and wild-type C57BL/6 splenocytes were used as a positive control. Expression was measured by surface staining with anti-MHC Class I (H-2Db) (clone 28-14-8, eBioscience) and anti-MHC Class I (H-2Kb) (clone AF6-88.5.5.3, eBioscience) according to manufacturer's protocols.

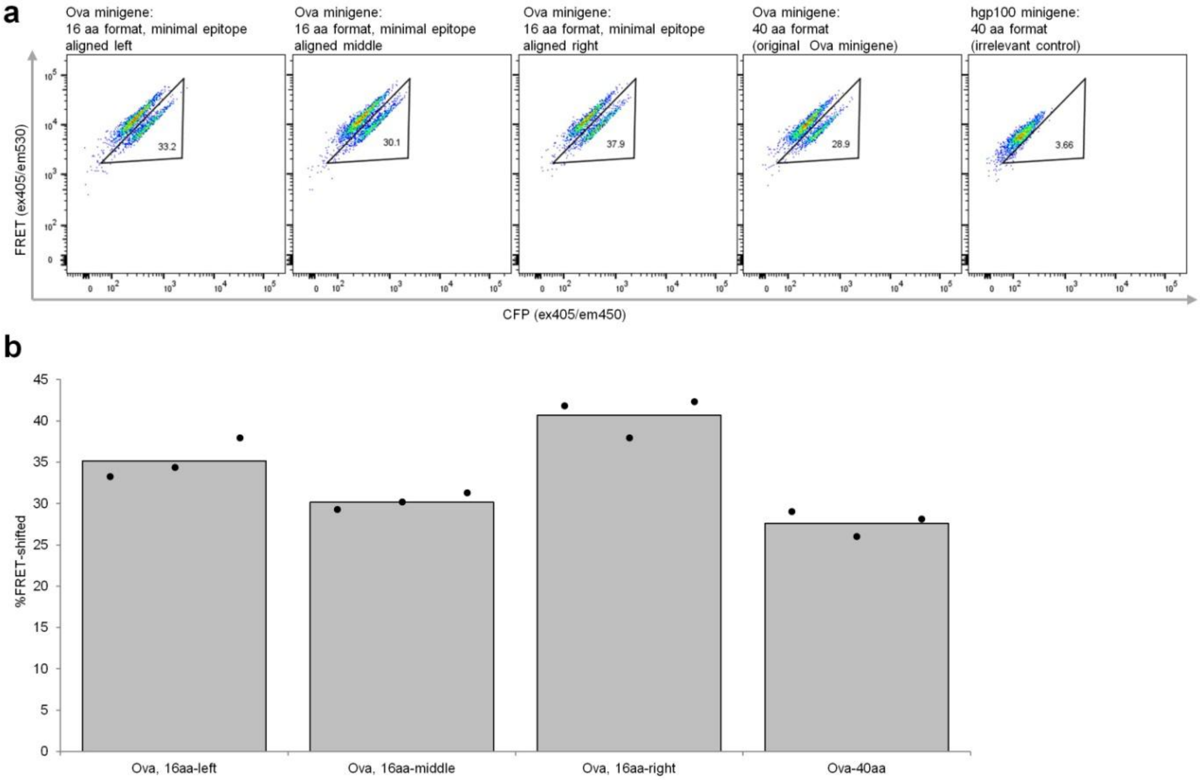

Supplementary Figure 7. Comparison of 40 amino acid and 16 amino acid format minigenes. Ova minigenes encoding peptides of length 16 were designed in three different configurations: with the SIINFEKL epitope oriented at the N-terminal end (extreme left) of the minigene, directly in the middle of the minigene, or at the C-terminal end (extreme right) of the minigene. EL4 cells expressing each of these configurations were produced by lentiviral transduction and were assessed by FRET-shift assay after co-culture with activated OT-I CTL. (a) Representative images of FRET-shift plots in flow cytometry for all Ova minigenes as well as an irrelevant minigene control. (b) All Ova minigene formats were conducted in sets of 3 technical replicates. All 16 amino acid format minigenes elicited slightly stronger FRET-shifts than the 40 amino acid minigenes, negating any concern that the longer Ova or hgp100 minigenes that were spiked into the shorter random minigene libraries may have had any advantage in screening experiments.

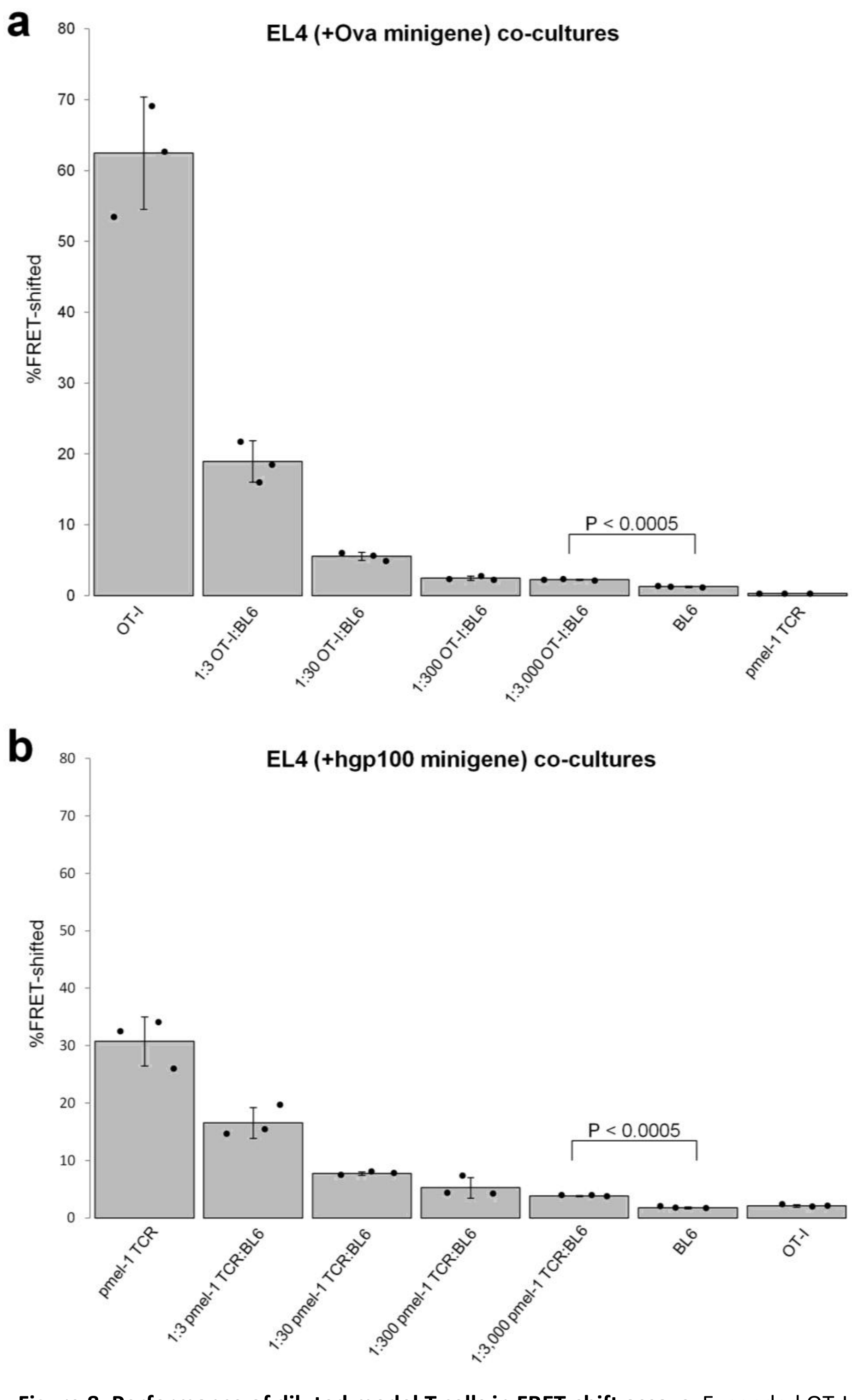

Supplementary Figure 8. Performance of diluted model T cells in FRET-shift assays. Expanded OT-I CTL and pmel-1 TCR CTL were each diluted into expanded wildtype C57BL/6 CTL at abundances ranging from 1:30 down to 1:3,000. OT-I and pmel-1 TCR cell mixtures were then co-cultured with pure, unmixed populations of EL4 cells transduced with Ova minigene and hgp100 minigene, respectively. Co-cultures of unmixed target cells and unmixed CTL were also performed as controls. Data shown are 3 replicate measurements with underlaid bar chart and error bars denoting mean ± SD. Significance was determined using an unpaired, 1-tailed Student's t test.

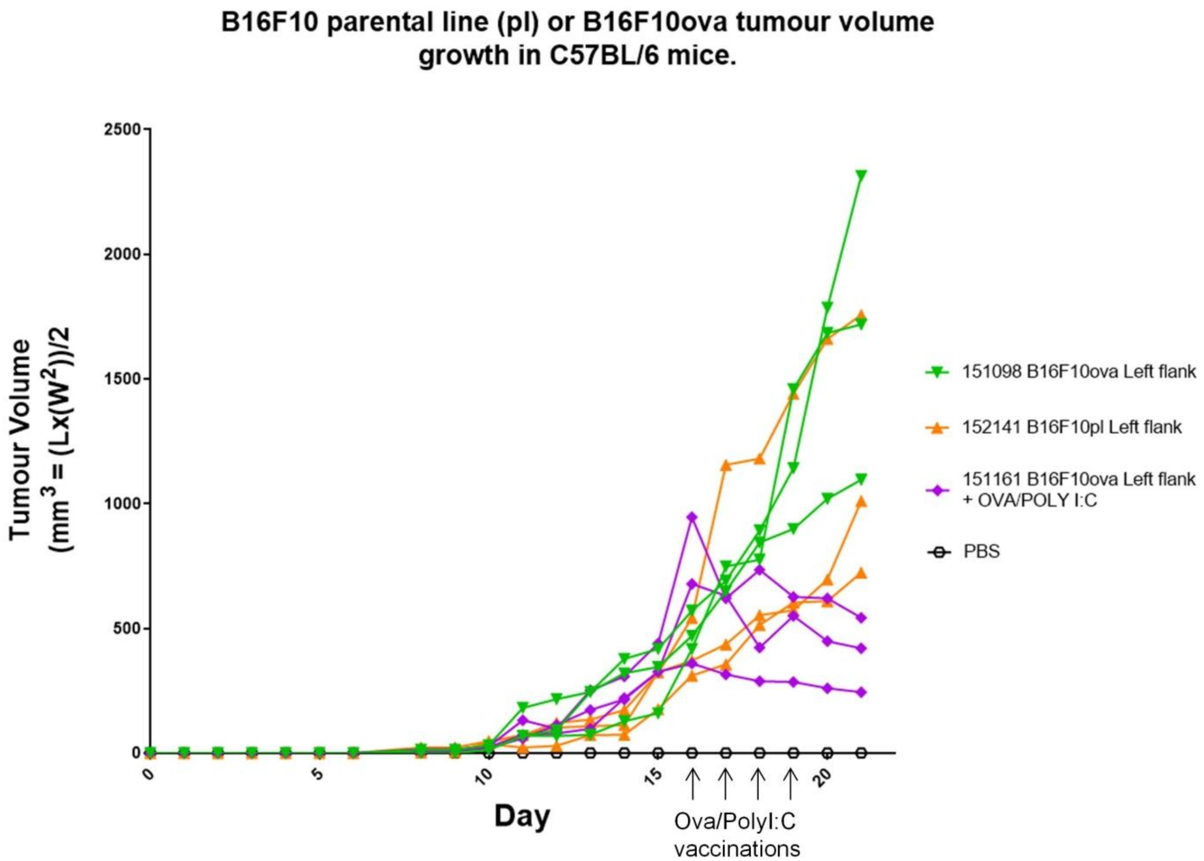

Supplementary Figure 9. B16F10 tumor grafts used as a source of test TIL for screening. Tumor growth curves of C57BL/6 male mice subcutaneously injected with B16F10pl (parental line) cells, B16F10-Ova (ovalbumin-expressing) cells with no vaccination, B16F10-Ova cells with Ova vaccine boost, or PBS only. A continued reduction in tumor volume was seen in the B16F10-Ova tumor graft mice when vaccinated, indicating that an Ova antigen-mediated anti-tumor response was occurring in these mice. Data shown are measurements of 3 replicate mice per group.

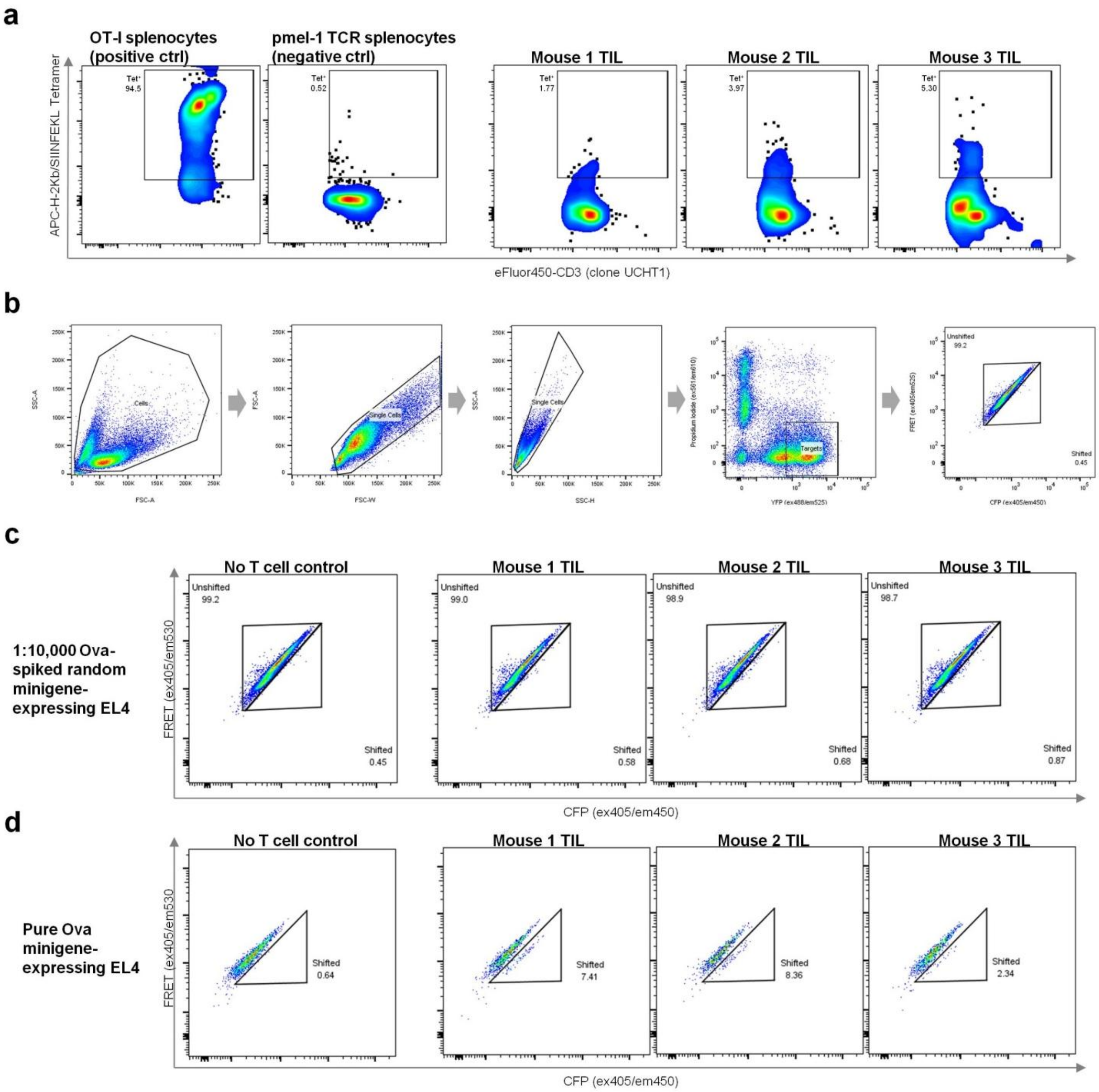

Supplementary Figure 10. Tetramer and FRET-shift characterizations of TIL screens. (a) Tetramer characterization of CD8+ TIL isolated from the tumors of each B16F10-Ova engrafted mouse. Using an APC-conjugated H-2kb-SIINFEKL tetramer (prepared by the NIH Tetramer Core Facility), we quantified the frequency of Ova-binding T cells in the CD8+ TIL population mediating the tumor regression noted in Suppl. Fig. 9. (b) The representative gating strategy used to isolate Shifted and Unshifted populations during FRET-shift FACS isolation prior to minigene recovery and sequencing. Cells were first gated on a series of forward scatter vs. side scatter plots to remove debris and isolate single cells. Live target cells were then gated using a combination of propidium iodide and YFP signal. FRET-signature was assessed by establishing an operator defined boundary in the FRET versus CFP plot of a T cell-unexposed control sample. (c) Images of FRET-shift plots used to isolate each individual sample reported in Figure 6. The appearance of a FRET-shift population is not apparent visually due to the rarity of cognate target cells in the 1:10,000 population. (d) Parallel controls conducted to assess the potency of TIL samples against unmixed populations of pure Ova minigene-expressing EL4 cells. The appearance of a FRET-shift population in response to T cell co-culture in these samples indicate that functional Ova-reactive T cells were present in the TIL population, confirming the tetramer observations from (a).

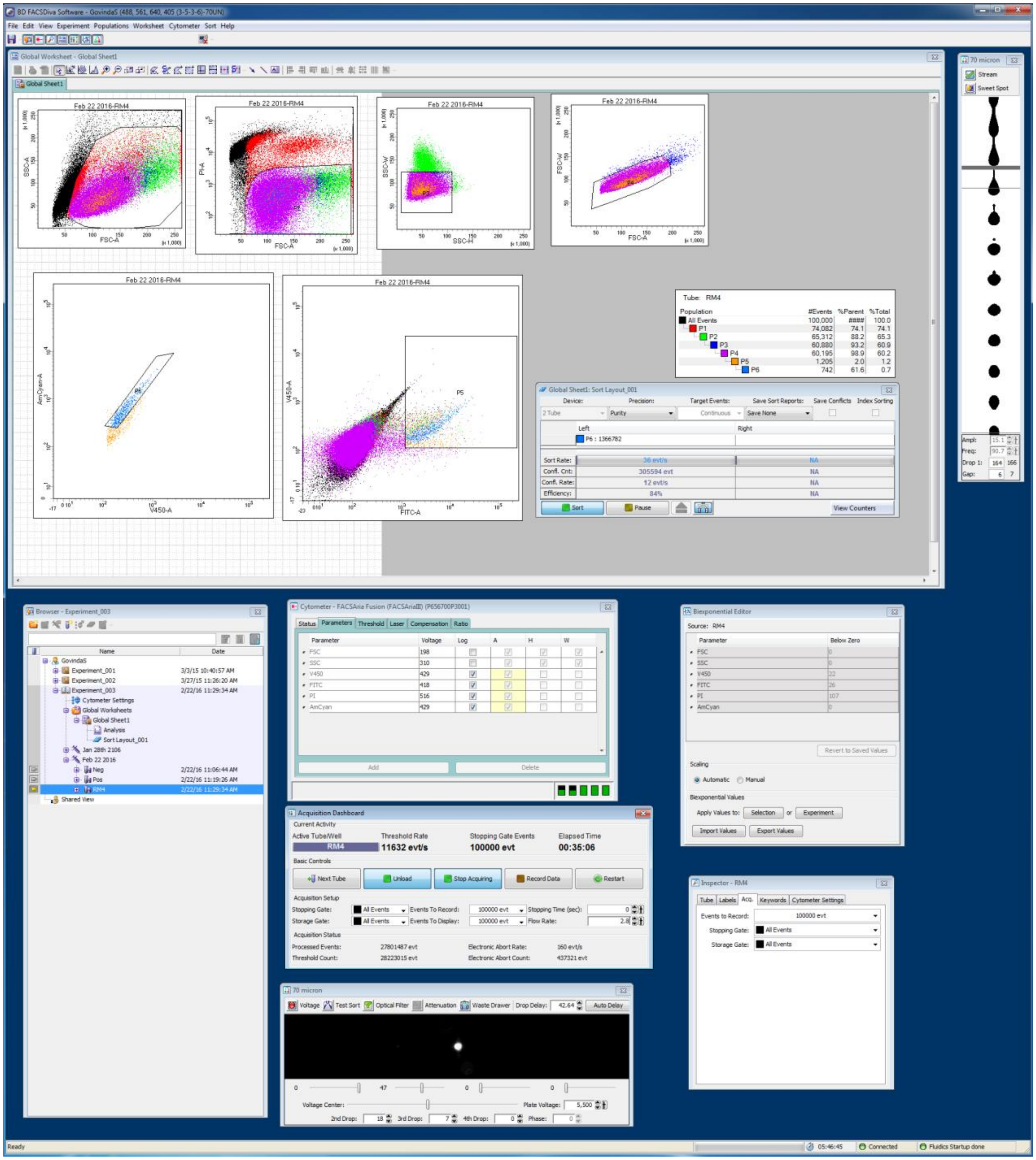

Supplementary Figure 11. Representative gating strategy for FRET purity sorting in target cells. Screenshot taken from BD FACSDiva software taken during a BD FACSAria Fusion target purity-sort. Displayed is the sequence of gates used to prepare pure populations of minigene-expressing ID8 or EL4 cells with clean resting FRET-signature suitable for use in FRET-shift assays. FRET^{+} gate is set by selecting stoichiometrically diagonal populations out of YFP^{+} populations. These populations can be visually determined as being parallel to autofluorescent cells in the negative control populations on FRET vs. CFP plot but shifted up in FRET detection channel.

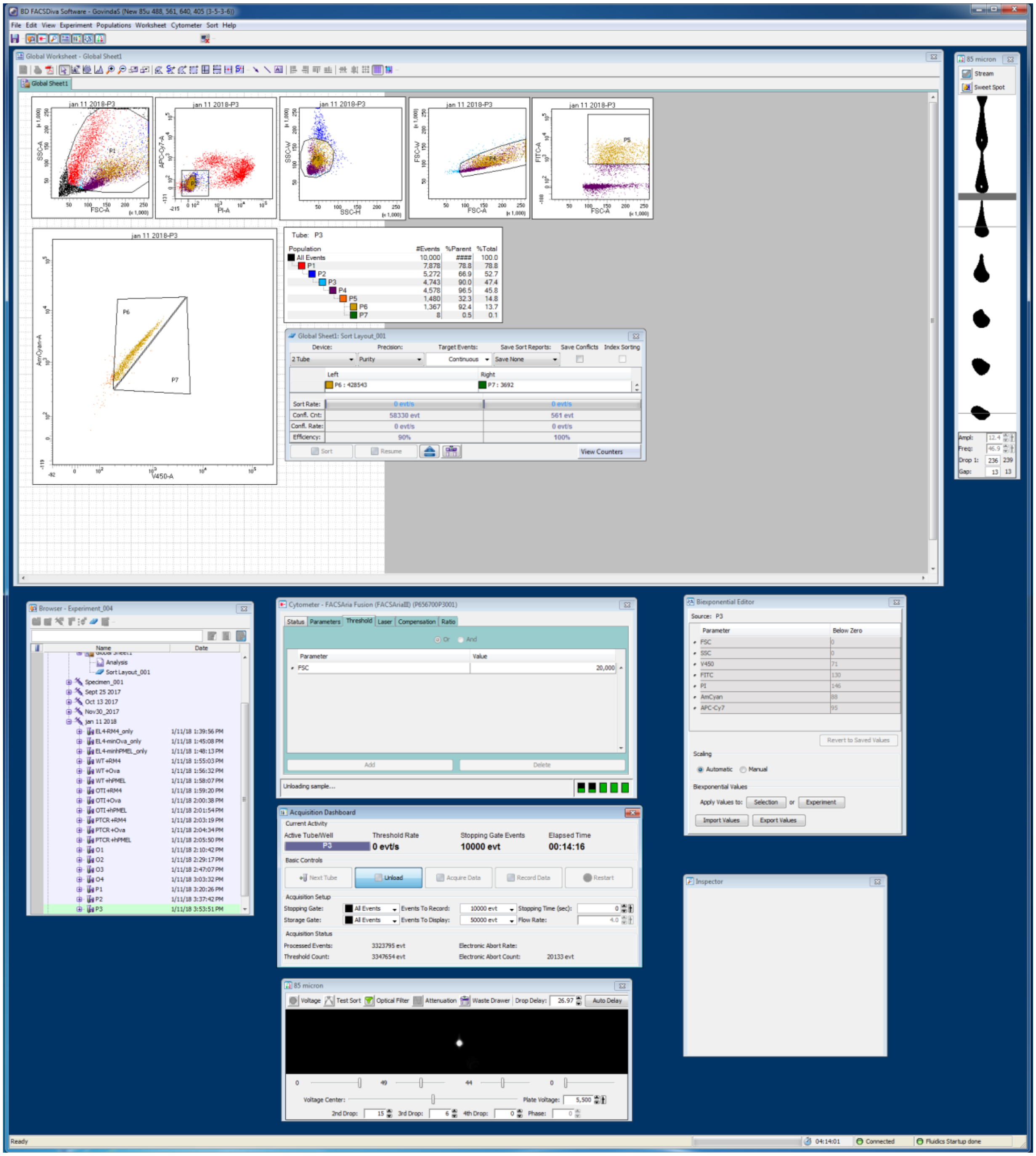

Supplementary Figure 12. Representative gating strategy for FRET-shift assay sorting in target cells. Screenshot taken from BD FACSDiva software taken during an experiment run on BD FACSAria Fusion. Shown is the sequence of gates used to perform 2-way sorting of minigene library-expressing ID8 or EL4 APC cell lines at the conclusion of co-cultures with effector cells to obtain Unshifted and Shifted target populations for downstream Illumina sequencing. Gates are set using no-effector APC control samples.

Supplementary Table 1. Construction of random minigene library expressed in ID8 cells. Random minigene library insert DNA was cloned into pMND-minigene-FRET plasmid backbone (Suppl. Fig. 1) and transformed into bacteria to yield 1.8x10^{6} cfu. Random minigene plasmid isolated from transformants was used to make random minigene lentivirus. Virus was functionally titered and delivered to host ID8 cells at an MOI of 0.36 TU/cell. Transduced ID8 were expanded 2.5-fold before FRET purity-sorting. The recovered cells were then expanded 6-fold further before being used in random minigene screening. After FRET-shift/amplicon sequencing, Lincoln-Peterson capture-recapture estimation was done by comparing the unique minigene sequences detected in all pairwise combinations of the six co-culture conditions sequenced in Fig. 3. The mean ± standard deviation of these estimates is reported below. It was estimated that 1.3x10^{6} unique minigenes were represented in the input APC population, indicating some loss in diversity in the final transduced target population relative to the plasmid-inserted minigene population.

**Step**	**PCR/ digestion/ purification**	**Ligation/ transformation**	**Virus production**	**Transduction**	**Target cell library prep/ CTL co-cultures**	**Sequencing/ Capture-Recapture estimation**
**Estimated library size**	•96 ng<br>•2.7 pmol	•1.8x10^{6} CFU	•1.9x10^{7} infectious units<br>•54% expected attrition due to stop codons	•MOI=0.36<br>•Expanded 2.5x prior to FACS	•1.1x10^{7} cells recovered from purity sorting<br>•Expanded 6x prior to cryopreservation<br>•3x10^{6} used per co-culture	•1.6x10^{6} filtered reads across 6 samples<br>•Estimated 1.34±0.03x10^{6} unique minigenes expressed by APC population

Supplementary Table 2. Construction of random minigene library expressed in EL4 cells. Random minigene plasmid described in Suppl. Tab. 1 was used to make a 2nd batch of random minigene lentivirus. Virus was functionally titered and delivered to host EL4 cells at an MOI of 0.11 TU/cell. Transduced EL4 were expanded 4-fold before FRET purity-sorting. The recovered cells were then expanded 125-fold further before being used in random minigene screening. After FRET-shift/amplicon sequencing, Lincoln-Peterson capture-recapture estimation was done by comparing the unique minigene sequences detected in all pairwise combinations of the seven co-culture conditions shown in Fig. 4a, Fig. 5. The mean ± standard deviation of these estimates is reported below. It was estimated that 4.7×10^5 unique minigenes were represented in the input APC population.

**Step**	**PCR/ digestion/ purification**	**Ligation/ transformation**	**Virus production**	**Transduction**	**Target cell library prep/ CTL co-cultures**	**Sequencing/ Capture-Recapture estimation**
**Estimated library size**	•96 ng<br>•2.7 pmol	•1.8×10^6 CFU	•1.1×10^7 infectious units<br>•54% expected attrition due to stop codons	•MOI=0.11<br>•Expanded 4x prior to FACS	•2×10^6 cells recovered from purity sorting<br>•Expanded 125x prior to cryopreservation<br>•3×10^6 used per co-culture	•6.7×10^6 filtered reads across 7 samples<br>•Estimated $4.68 \pm 0.2 \times 10^5$ unique minigenes expressed by APC population

Supplementary Table 3. Database searching of random minigene hits from 1:10,000 spiked samples. The 2 random minigene hits from OT-I screening meeting 10σ cutoff and 14 random minigene hits meeting the 10σ cutoff and being more highly enriched than the hgp100 minigene in pmel-1 TCR screening were subject to peptide prediction using NetMHCpan 3.0 and BLASTP alignment to all non-redundant GenBank CDS translations+PDB+SwissProt+PIR+PRF excluding environmental samples from WGS projects.

**Test TCR**	**Rank in screen**	**Encoded minigene**	**Minimal peptides with predicted IC50 < 500nM?**	**Top E-score from BLASTp alignment**
**OT-I**	1	NTAKTTGKPSVVQATM	No	22
**OT-I**	2	HIHTDWPQYPPCLNLI	No	1.8
**OT-I**	3	MLVLLPDEVSGLEQLE**SIINFEKL**TEWTSSNVMEERKIKV	Yes, predicted IC50 = 44.40 nM	1.4×10^{-18}
**pmel-1 TCR**	1	QILLPIHKHITTLVGA	No	11
**pmel-1 TCR**	2	SNADVQHSTIRPQPHT	No	5.7
**pmel-1 TCR**	3	SKNKPLLNGVTCIMSK	No	11
**pmel-1 TCR**	4	STRYSGTVLYLVVQRR	No	2.0
**pmel-1 TCR**	5	DAHCGNVEPTTKTLNL	No	8.1
**pmel-1 TCR**	6	EHEVTLDGTIKATLAK	No	5.7
**pmel-1 TCR**	7	FKFTILKRSTIESIH	No	14
**pmel-1 TCR**	8	LSCPCQNLLPHVSFPR	No	2.0
**pmel-1 TCR**	9	QYGRTPKGPFLLTAPK	No	2.9
**pmel-1 TCR**	10	REKVYMPPILKHGPDN	Yes, predicted IC50 = 409.90 nM	2.0
**pmel-1 TCR**	11	RQNQHFALHKNESKKD	No	1.0
**pmel-1 TCR**	12	RTHKNNLNTTDAFPLI	No	2.0
**pmel-1 TCR**	13	TVKFRPPPPAVWTDPR	No	0.25
**pmel-1 TCR**	14	NLRNITHAGGRITPH	No	2.4
**pmel-1 TCR**	15	LLHLAVIGALLAVGAT**KVPRNQDWL**GVSRQLRTKAWNRQLY	Yes, predicted IC50 = 123.00 nM	3.0×10^{-19}

Supplementary table 4. A list of relevant oligonucleotide sequences used. A summary of the synthesized oligonucleotides used to generate Ova minigene, Ova scrambled-epitope minigene, hgp100 minigene, and random minigenes as well as the primers needed to generate Illumina adapterized sequencing libraries from collected minigenes and to enumerate viral integrations per sample by qPCR. All oligonucleotides were sourced from Integrated DNA Technologies.

**Oligo name**	**Oligo description**	**Oligo sequence (5'-3')**
Ova(241-280)_FWD	Forward primer for amplifying Ova minigene from OVAL (*Gallus gallus*) coding sequence. Tailed with BamHI site.	ATGCGGATCCATGTTGGTGCTGT TGCCT
Ova(241-280)_REV	Reverse primer for amplifying Ova minigene from OVAL (*Gallus gallus*) coding sequence. Tailed with EcoRI site.	ATGCGAATTCCACTTTGATCTTC CTCTCTTCC
Ova(241-280|SCR)_FWD	Forward overlap extension oligo for constructing scrambled-epitope Ova minigene containing scrambled epitope (LKNFISEI). Tailed with a BamHI restriction site.	ATGCGGATCCATGTTGGTGCTGT TGCCTGATGAAGTCTCAGGCCTT GAGCAGCTTGAGCTGAAAAACT TTATCAGTGAAA
Ova(241-280|SCR)_REV	Reverse overlap extension oligo for constructing Ova minigene containing scrambled epitope (LKNFISEI). Tailed with an EcoRI restriction site.	ATGCGAATTCCACTTTGATCTTC CTCTCTTCCATAACATTAGAACT GGTCCATTCAGTTATTTCACTGA TAAAGTTTTTC
hgp100(9-59)_FWD	Forward overlap extension oligo for constructing human gp100 minigene encoding amino acid position 9 to 49. Tailed with a BamHI restriction site.	ATGCGGATCCCTTCTTCATTTGG CTGTGATAGGTGCTTTGCTGGCT GTGGGGGCTACAAAAGTACCCA GAAACCAGGACT
hgp100(9-59)_REV	Reverse overlap extension oligo for constructing human gp100 minigene encoding amino acid position 9 to 49. Tailed with an EcoRI restriction site.	CATGGAATTCATACAGCTGCCTG TTCCAGGCTTTGGTTCTGAGTTG CCTTGAGACACCAAGCCAGTCCT GGTTTCTGGGTAC
RM_FWD	Forward primer for random minigene library amplification. Tailed with arbitrary sequence to facilitate resolution of cut and uncut fragments on agarose gels.	CGTAGTTATCCTGTATCGGATGA GAATTCTGCATCGGGCCAGCCA CGTTTGGTGGAATTC
RM_REV	Reverse primer for random minigene library amplification. Tailed with arbitrary sequence to facilitate resolution of cut and uncut fragments on agarose gels	CTGTACTAATAGCACACACGGG GGATTCCCAGCACAAGCTAGTC ATGCAGCTCCGGATCC
RM_template	Degenerate oligo for construction of minigene library. Encodes BamHI/EcoRI restriction sites immediately flanking degenerate regions.	CCACGTTTGGTGGAATTCNNNNN NNNNNNNNNNNNNNNNNNN NNNNNNNNNNNNNNNNNNN NNNNNNGGATCCGGAGCTGCA T
MND25_FWD	Forward primer for qPCR detection of integrated viral cassettes.	GCAAGCTAGGATCAAGGTTAGG
MND148_REV	Reverse primer for qPCR detection of integrated viral cassettes.	TGGCCCATATTCTGCTGTTC
Minigene_Illumina_FWD	Illumina-adapterized forward PCR primer for single round amplicon library preparation and direct minigene sequencing	AATGATACGGCGACCACCGAGA TCTACACTCTTTCCCTACACGAC GCTCTTCCGATCTCTGGTTCGCT TCTCGCTTCTGTT

**Supplementary table 4. A list of relevant oligonucleotide sequences used (continued)**		
**Oligo name**	**Oligo description**	**Oligo sequence (5'-3')**
Minigene_Illumina+index_REV	Illumina-adapterized reverse PCR primer for single round amplicon library preparation and direct minigene sequencing	CAAGCAGAAGACGGCATACGAGATNNNNNNGTGACTGGAGTTCAGACGTGTGCTCTTCCGATCTGACGTTAGTAGCTCCGCTTCC
Minigene_FWD _IA+stagger	Forward primer for amplifying minigenes from recovered gDNA in the 1st round of 2-round PCR library preparation scheme. Tailed with annealing region for 2nd round primers containing complete Illumina adapter sequence. Composed of an equimolar mix of oligos containing 1-9 arbitrary staggering bases located between minigene annealing region and Illumina adapter region.	CGCTCTTCCGATCTCTG$(N)_{1-9}$GTTCGCTTCTCGCTTCTGTT
Minigene_REV_IA+stagger	Reverse primer for amplifying minigenes from recovered gDNA in the 1st round of 2-round PCR library preparation scheme. Tailed with annealing region for 2nd round primers containing complete Illumina adapter sequence. Composed of an equimolar mix of oligos containing 1-9 arbitrary staggering bases located between minigene annealing region and Illumina adapter region.	TGCTCTTCCGATCTGAC$(N)_{1-9}$GTTAGTAGCTCCGCTTCC
Illumina_FWD	Forward primer for 2nd round PCR in Illumina library preparation.	AATGATACGGCGACCACCGAGATCTACACTCTTTCCCTACACGACGCTCTTCCGATCTCTG
Illumina_REV	Reverse primer for 2nd round PCR in Illumina library preparation. Contains variable index sequence (underlined).	CAAGCAGAAGACGGCATACGAGATNNNNNNGTGACTGGAGTTCAGACGTGTGCTCTTCCGATCTGAC
