# Supplementary material for: Rapid selection and identification of functional CD8+ T cell epitopes from large peptide-coding libraries
Source: Nat Commun. 2019 Oct 7;10:4553. doi: 10.1038/s41467-019-12444-7 (PMC6779888; doi:10.1038/s41467-019-12444-7)
Supplement: Supplementary file 3 — Reporting Summary [file 41467_2019_12444_MOESM3_ESM.pdf]

nature research

Corresponding author(s): Robert Holt

☐ Initial submission ☒ Revised version ☐ Final submission

Life Sciences Reporting Summary

Nature Research wishes to improve the reproducibility of the work that we publish. This form is intended for publication with all accepted life science papers and provides structure for consistency and transparency in reporting. Every life science submission will use this form; some list items might not apply to an individual manuscript, but all fields must be completed for clarity.

For further information on the points included in this form, see Reporting Life Sciences Research. For further information on Nature Research policies, including our data availability policy, see Authors & Referees and the Editorial Policy Checklist.

Experimental design

1. Sample size

Describe how sample size was determined.

No statistical methods were used to predetermine the sample size. The number of cells input into individual co-cultures and number of co-cultures per experiment were based on practical considerations including the yield of T-cell expansion from starting splenocytes and limitations on FACS instrument time available to sort experimental samples.

2. Data exclusions

Describe any data exclusions.

In antigen identification screens using Ova protein (+ polyIC adjuvant) vaccinated mice versus polyIC-only vaccinated mice we found that the Ova antigen was detected by T cells from spleens from both groups. Subsequent staining with a SIINFEKL/H-2Kb tetramer sourced from the NIH tetramer core facility revealed, unexpectedly, the presence of Ova tetramer+ T cells in the polyIC treated mice. We excluded these data because the experiment lacks an appropriate control, and further work to try to explain the observation is beyond our current scope.

3. Replication

Describe whether the experimental findings were reliably reproduced.

During development, some experiments failed due to environmental contamination, which was readily recognized and rectified. All other FRET-shift/amplicon sequencing experiments were reliably reproducible.

4. Randomization

Describe how samples/organisms/participants were allocated into experimental groups.

Animals were randomly allocated to experimental groups.

5. Blinding

Describe whether the investigators were blinded to group allocation during data collection and/or analysis.

FACS was conducted by British Columbia Cancer Research Centre core facility technicians guided by a pre-defined sort template provided by the first author and were blinded to sample identities/groups. Sequencing was performed at a separate facility of the British Columbia Cancer Research Centre by technicians who were also blinded to sample identities/groups.

Note: all studies involving animals and/or human research participants must disclose whether blinding and randomization were used.

6. Statistical parameters

For all figures and tables that use statistical methods, confirm that the following items are present in relevant figure legends (or in the Methods section if additional space is needed).

n/a	Confirmed	
☐	☒	The exact sample size (*n*) for each experimental group/condition, given as a discrete number and unit of measurement (animals, litters, cultures, etc.)
☐	☒	A description of how samples were collected, noting whether measurements were taken from distinct samples or whether the same sample was measured repeatedly
☐	☒	A statement indicating how many times each experiment was replicated
☐	☒	The statistical test(s) used and whether they are one- or two-sided (note: only common tests should be described solely by name; more complex techniques should be described in the Methods section)
☐	☒	A description of any assumptions or corrections, such as an adjustment for multiple comparisons
☐	☒	The test results (e.g. *P* values) given as exact values whenever possible and with confidence intervals noted
☐	☒	A clear description of statistics including central tendency (e.g. median, mean) and variation (e.g. standard deviation, interquartile range)
☐	☒	Clearly defined error bars

See the web collection on statistics for biologists for further resources and guidance.

Software

Policy information about availability of computer code

7. Software

Describe the software used to analyze the data in this study.	ImageQuant TL v8.1, FlowJo V10.0.8r1, SDS2.4, BD FACSDiva V8.0.1, Geneious v8.1.2, R v3.1.2, RStudio, FASTx-toolkit, FLASh, Starcode

For manuscripts utilizing custom algorithms or software that are central to the paper but not yet described in the published literature, software must be made available to editors and reviewers upon request. We strongly encourage code deposition in a community repository (e.g. GitHub). *Nature Methods* guidance for providing algorithms and software for publication provides further information on this topic.

Materials and reagents

Policy information about availability of materials

8. Materials availability

Indicate whether there are restrictions on availability of unique materials or if these materials are only available for distribution by a for-profit company.	Materials are available at cost through a Material Transfer Agreement with the British Columbia Cancer Research Centre

9. Antibodies

Describe the antibodies used and how they were validated for use in the system under study (i.e. assay and species).	For T-cell activation:<br><br>Biolegend 100314: LEAF-purified anti-mouse CD3e, clone 145-2C11 [https://www.biolegend.com/en-us/search-results/leaf-low-endotoxin--azide-freepurified-anti-mouse-cd3epsilon-antibody-24]<br><br>Biolegend 102112: LEAF-purified anti-mouse CD28, clone 37.51 [https://www.biolegend.com/en-us/products/leaf-low-endotoxin--azide-freepurified-anti-mouse-cd28-antibody-114]<br><br>For cell phenotyping:<br><br>ebioscience 46-5958-80: PerCP-eFluor710 conjugated anti-mouse MHC Class I (H-2Kb), clone AF6-88.5.5.3 [https://www.thermofisher.com/antibody/product/MHC-Class-I-H-2Kb-Antibody-Monoclonal/46-5958-80]<br><br>ebioscience 17-5999-80: APC conjugated anti-mouse MHC Class I (H-2Db), clone 28-14-8 [https://www.thermofisher.com/antibody/product/MHC-Class-I-H-2Db-Antibody-clone-28-14-8-Monoclonal/17-5999-80]<br><br>ebioscience 25-5743-80: PE-Cy7 conjugated anti-mouse H-2Kb/OVA257-264 complex, clone 25-D1.16 [https://www.thermofisher.com/antibody/product/OVA257-264-SIINFEKL-peptide-bound-to-H-2Kb-Antibody-clone-eBio25-D1-16-25-D1-16-Monoclonal/25-5743-80]<br><br>ebioscience 61-0031-80: PE-eFluor610 conjugated anti-mouse CD3e, clone 145-2C11 [https://www.thermofisher.com/antibody/product/CD3e-Antibody-clone-145-2C11-Monoclonal/61-0031-80]<br><br>ebioscience 47-0081-82: APC-eFluor780 conjugated anti-mouse CD8a, clone 53-6.7 [https://www.thermofisher.com/antibody/product/CD8a-Antibody-clone-53-6-7-Monoclonal/47-0081-82]<br><br>H-2Kb/SIINFEKL (human b2m) APC-labeled tetramer was obtained from the NIH Tetramer Core Facility

10. Eukaryotic cell lines

a. State the source of each eukaryotic cell line used.	ID8 and EL4 cell lines were sourced from the BCCA Deeley Research Centre, Victoria, BC, Canada. B16F10 cells were purchased from ATCC.
b. Describe the method of cell line authentication used.	No cell line authentication was performed.
c. Report whether the cell lines were tested for mycoplasma contamination.	All cell lines were tested for mycoplasma contamination using Venor GeM mycoplasma testing kit (Sigma). Only cell lines testing negative were used in experiments.
d. If any of the cell lines used are listed in the database of commonly misidentified cell lines maintained by ICLAC, provide a scientific rationale for their use.	No commonly misidentified cell lines were used.

Animals and human research participants

Policy information about studies involving animals; when reporting animal research, follow the ARRIVE guidelines

11. Description of research animals

Provide details on animals and/or animal-derived materials used in the study.	OT-I splenocytes were sourced from BCCA Deeley Research Centre, Victoria, BC, Canada.<br><br>pmel-1 TCR spleens were sourced from The Jackson Laboratory, Bar Harbor, ME, USA. Splenocytes were isolated in-house<br><br>Wild-type male C57BL/6 mice, aged between 4 to 5 weeks of age, were bred and housed at the BC Cancer Research Centre's Animal Resource Centre for the duration of the study. Protocols involving mouse tumor graft experiments were reviewed for ethics compliance and approved by the University of British Columbia Animal Care Committee (A18-0197)

Policy information about studies involving human research participants

12. Description of human research participants

Describe the covariate-relevant population characteristics of the human research participants.	Study did not involve human research participants
