# Supplementary material for: Rapid selection and identification of functional CD8+ T cell epitopes from large peptide-coding libraries
Source: Nat Commun. 2019 Oct 7;10:4553. doi: 10.1038/s41467-019-12444-7 (PMC6779888; doi:10.1038/s41467-019-12444-7)
Supplement: Supplementary file 2 — Peer Review file [file 41467_2019_12444_MOESM2_ESM.pdf]

Editorial Note: This manuscript has been previously reviewed at another journal that is not operating a transparent peer review scheme. The manuscript was considered suitable for publication without further review at Nature Communications.
